# Recent Advances in Anticancer Activity of Gold(I) Complexes

**DOI:** 10.3390/biomedicines14071562

**Published:** 2026-07-12

**Authors:** Nikhil Bhimsing Khandale, Jitendra Gour, Iqubal Singh, Chandan Bhogendra Jha, Avani Farasrami, Neeraj Kumar Chouhan

**Affiliations:** 1 Department of Pharmaceutical Chemistry, Nims Institute of Pharmacy, Nims University Rajasthan, Jaipur 303121, Rajasthan, India; nikhil.khandale@nimsuniversity.org; 2 Department of Chemical Sciences, National Institute of Pharmaceutical Education and Research (NIPER), Hyderabad 500037, Telangana, India; jeetugour1810@gmail.com; 3 School of Pharmaceutical Sciences, Lovely Professional University, Phagwara 144411, Punjab, India; 4 Department of Chemical Engineering, Indian Institute of Technology Roorkee, Roorkee 247667, Uttarakhand, India; 5 Flamma USA LLC, 383 Phoenixville Pike, Malvern, PA 19355, USA

**Keywords:** anticancer, donor ligands, gold(I) complex, in vitro studies, thioredoxin reductase

## Abstract

The clinical success of cisplatin has significantly spurred the exploration of new organometallic complexes in oncology. In this quest, repurposing of auranofin as an anticancer agent has diverted the research interest from platinum to gold complexes, as gold offers unique chemical features; among them, thioredoxin reductase (TrxR) inhibition is one of the most extensively studied anticancer pathways. In this study, we have compiled the major ligand modifications reported for gold(I) complexes and categorized them into various groups, which include sulfur-based ligands, nitrogen-containing heterocyclic ligands, carbon-derived ligands, and *N*-heterocyclic carbene-based ligands. Also, a few structurally distinct ligands, including propargyl-, allene-, tricarbene-, and urea-functionalized NHC frameworks, have further extended structural diversity and functional potential. The in vitro evaluation of these newly synthesized gold complexes against various cancer cell lines exhibited enhanced biological potential compared to conventional metal complexes. Comparative evaluation of the reported cytotoxicity data revealed distinct structure–activity relationships among different ligand classes, with phosphine-carbon donor and bis-NHC frameworks emerging as the most promising ligand for achieving potent anticancer activity, highlighting the critical role of ligand design in modulating anticancer activity. In addition, the use of bioactive pharmacophores derived from natural products and active pharmaceuticals has emerged as a promising design strategy for developing multitarget gold(I) complexes with enhanced therapeutic efficacy. Among the reviewed compounds, complex **68** containing a bis-NHC ligand exhibited the highest potency against HL-60 leukemia cells (GI_50_ = 0.017 μM), while complex **49** bearing a carbon-donor ligand demonstrated remarkable activity against A549 lung cancer cells (IC_50_ = 0.02 μM). Several other gold(I) complexes also exhibited submicromolar activity against diverse cancer cell lines, further emphasizing the importance of rational ligand engineering in enhancing anticancer efficacy. Collectively, gold(I) complexes have emerged as a promising class of anticancer agents, and the comparative evaluation presented herein provides a valuable framework for identifying potent ligand scaffolds and guiding the rational development of next-generation gold-based therapeutics. Future advances in ligand engineering may facilitate targeted drug delivery, controlled release, and multi-mechanistic therapeutic strategies to overcome toxicity and drug resistance while enhancing therapeutic efficacy.

## 1. Introduction

**Figure 1:**
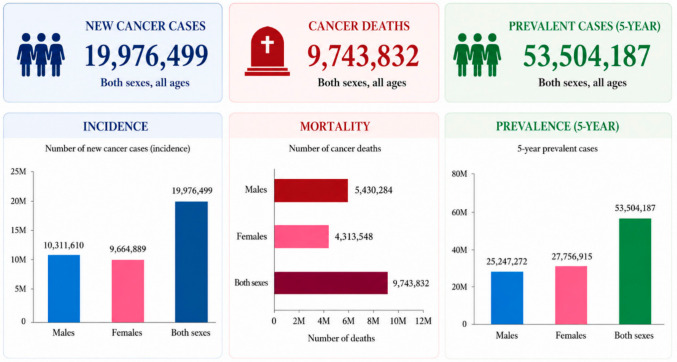
This figure summarizes the global cancer burden and epidemiological profile in 2022. Total reported cases, deaths, and 5-year prevalence, showing higher mortality in males and greater prevalence in females. Overall, this visual provides an integrated overview of global patterns, sex-based differences, and the relative contribution of major cancer types to the total burden.

Cancer is described as a group of diseases posing a serious global health problem by causing millions of deaths every year. This noncommunicable disease develops due to uncontrolled cell division, followed by the formation of clusters of tissue (tumors). Metastasis, a key hallmark of cancer marked by the migration of cancer cells from the origin site to other parts of the body, is primarily contributing to cancer-associated morbidity and mortality [1,2]. As per the World Health Organization’s latest survey, 20 million new cases and 9.7 million deaths occurred in 2022, and it is estimated to increase to more than 35 million by 2050. According to estimates, there are 225,000 cancer cases annually diagnosed in the US, and 160,000 people pass away from the disease (Figure 1) [3,4,5,6].

Treatment of cancer is still a major problem for the medical fraternity because of different types of cancer and different reasons of occurrence, and huge differences in the patient’s physical tolerances toward the drugs used [7,8]. Sometimes, the body can develop a resistance towards the present anticancer drugs, and this has posed a serious issue in the treatment of cancer [9]. Currently, various types of drug molecules are in practice for the treatment of cancer. Many small heterocyclic moieties have anticancer properties and are used for curing several types of tumors [10]. Additionally, complexes of metals with small heterocyclic moieties have promising cytotoxicity against various cancer cell lines. Commonly used commercial complexes are platinum (II)-based metallo drugs, such as cisplatin, carboplatin, and oxaliplatin, which have been used successfully to treat cancer [11,12]. However, the range of activity against various cancer types is constrained for such medications. The adverse effects of platinum (II) anticancer medications, such as neurotoxicity [13,14], ototoxicity [15,16], anemia [17,18], and nausea [19,20], as well as the acquired and inherent resistances displayed by cancer cell lines [21], primarily hinder the effectiveness of these medications. These detrimental and constrictive characteristics of platinum (II) medications are significant barriers to their therapeutic usage against all types of malignancies [22]. Consequently, non-platinum metal complexes, such as gold, have been developed and studied for their potential to enhance antitumor effect by combining greatly reduced adverse effects and toxicity to numerous cancer cell types. Metal ions with biological activity, such as silver (Ag) [23], gold (Au) [23], ruthenium (Ru) [24], rhodium (Rh) [25], Iridium (Ir) [26], zinc (Zn) [27], copper (Cu) [28] platinum (Pt) [29], palladium (Pd) [30], etc., are used to build metal complexes as anticancer agents. Many researchers are interested in studying complexes of gold with functional ligands because of the fascinating pharmacological, chemical, biological, and physical properties they show [31,32,33,34].

Gold(I) complexes have attracted considerable attention as potential anticancer agents due to their favorable coordination chemistry, structural versatility, and mechanisms of action that differ from those of conventional platinum-based drugs [35,36]. The therapeutic potential of gold(I) complexes can be significantly influenced by ligand design, which governs their stability, cellular uptake, target selectivity, and biological activity. Consequently, a broad range of ligand systems, including sulfur-based ligands, nitrogen-containing heterocyclic ligands, carbon-derived ligands, and *N*-heterocyclic carbene (NHC) frameworks, have been explored for the development of novel gold(I)-based anticancer agents [37,38]. Although several reviews have summarized the biological and mechanistic aspects of gold-based anticancer compounds [39], a comprehensive comparison of structurally diverse gold(I) complexes and their anticancer activities remains limited. Therefore, the present review systematically compiles gold(I) complexes and their subclasses reported in the literature and provides a comparative assessment of their cytotoxic activities against various cancer cell lines. Particular emphasis is placed on the comparison of reported IC_50_ values, along with SAR study, to identify the most promising ligand frameworks and gold(I) scaffolds for specific cancer types. By integrating structural information with biological performance, this review aims to provide researchers with a practical reference for the rational design and further development of next-generation gold(I)-based anticancer therapeutics.

## 2. Gold (I) Complexes as Emerging Anticancer Agents: Mechanisms of Action

**Figure 2:**
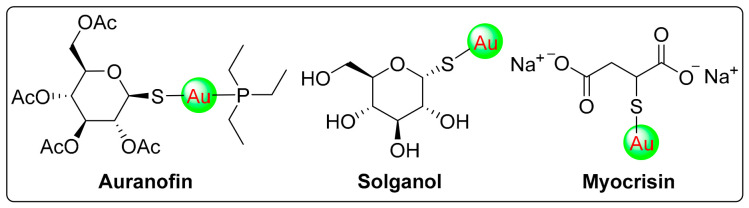
Currently prescribed gold medications.

Gold-derived compounds, particularly gold(I) complexes, have emerged as potential candidates in oncology due to their distinctive modes of action and selectivity against different in vitro models [40]. Beyond their direct cytotoxic effects, recent studies have demonstrated that selected gold(I) complexes can beneficially modulate the gut microbiome by selectively suppressing pathogenic bacteria while preserving beneficial commensal species, thereby enhancing anticancer efficacy and reducing treatment-associated gastrointestinal dysbiosis. These findings further expand the therapeutic potential of gold(I)-based agents as multifunctional anticancer drugs [41]. The clinical evidence of these compounds is proven by auranofin, an FDA-approved drug for rheumatoid arthritis, which has also demonstrated significant anticancer properties (Figure 2) [42,43]. Furthermore, the growing repertoire of gold(I) and gold(III) complexes exhibiting potent antiproliferative activity, together with their unique ability to target proteins and enzymes rather than DNA, has led to the advancement of several candidates into preclinical and clinical studies, reinforcing their promise as next-generation metal-based anticancer therapeutics [44].

The biological activity of gold complexes is strongly driven by the chemical nature of their ligands, which can be strategically tailored to optimize pharmacological properties. Till now, several types of ligands have been investigated, including phosphines, thiolates, dithiocarbamates, and nitrogen-containing heterocycles. These ligand systems impart stability, cellular uptake, and site specificity to the gold complexes [45,46]. Among them, gold(I) phosphine complexes are extensively studied due to their membrane permeability and intracellular delivery [47]. Analogously, thiolate and dithiocarbamate ligands have demonstrated promising biological profiles, specifically in altering redox balance and enzyme activity [48,49]. Further, nitrogen-donor ligands expand the structural diversity and functional adaptability of these complexes [50].

**Figure 3:**
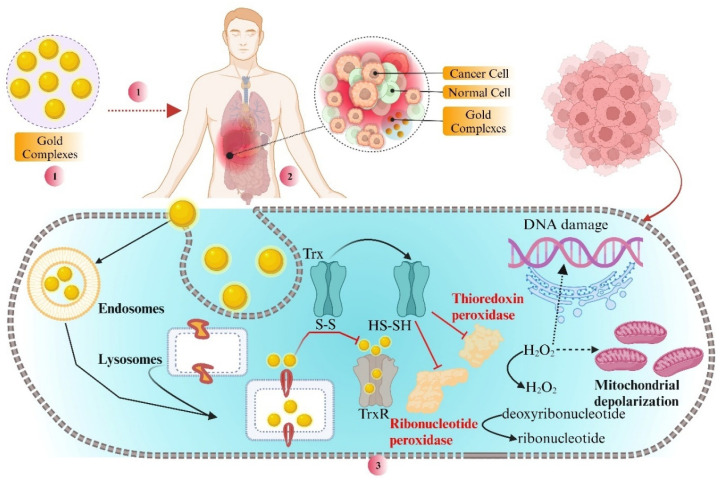
Targeting mitochondrial-mediated apoptosis: insights from gold complex pathway diagrams.

By receptor-mediated endocytosis, gold complexes enter lysosomes. After degradation in lysosomes, they release the gold ions. These gold ions bind with thioredoxin reductase (TrxR), particularly at thiol and selenol-containing active sites of TrxR, resulting in inhibition of its activity. Thus, it impairs cellular redox homeostasis and promotes the accumulation of both cytosolic and mitochondrial reactive oxygen species (ROS). This increased ROS levels induce mitochondrial dysfunction, followed by loss of mitochondrial membrane potential, impaired ATP synthesis, and activation of intrinsic apoptotic pathways. Additionally, oxidative stress and mitochondrial damage interfere with DNA repair mechanisms, thereby amplifying cellular damage. The inhibition of TrxR and subsequent ROS generation are well-established as key contributors to the cytotoxic effects of gold complexes in cancer cells (Figure 3) [51,52,53].

The present study discusses earlier reported gold(I) complexes with their anticancer activities. These structurally diverse complexes, possessing a range of ligand architectures, may provide valuable insights for the rational design and development of potent anticancer agents.

## 3. Phosphine-Based Gold(I) Complexes

Phosphorus-based ligands have been widely employed in the design of metal complexes, particularly gold(I), due to their promising therapeutic potential. In particular, gold(I) complexes with phosphorus-donor ligands have shown significant potential, as they can stabilize the gold(I) center. The stability of the gold(I) complex can be rationalized by Pearson’s Hard and Soft Acids and Bases (HSAB) theory. According to HSAB theory, the gold(I) center acts as a soft Lewis acid, and phosphine ligands behave as soft Lewis bases. Thus, both species possess a strong attractive force against each other and form a stable gold–phosphorus bond, which stabilizes gold in its +1-oxidation state. Furthermore, phosphorus ligand also improves reactivity, selectivity, and accessibility to alter solubility and lipophilicity via an appropriate ligand framework. Most frequently used phosphorus ligands include triphenylphosphine (PPh_3_), triethyl phosphine (PEt_3_), and tri-o-tolyl phosphine [P(o-tolyl)_3_]. Additionally, bidentate phosphorus ligands are also incorporated as linkages in binuclear gold(I) complexes to control their steric and electronic properties [54,55,56]. In this section, we have further categorized and discussed in detail various phosphorus donor ligands along with their anticancer effects on different cancer cell lines.

### 3.1. Phosphine-Sulfur Donor Complexes

**Figure 4:**
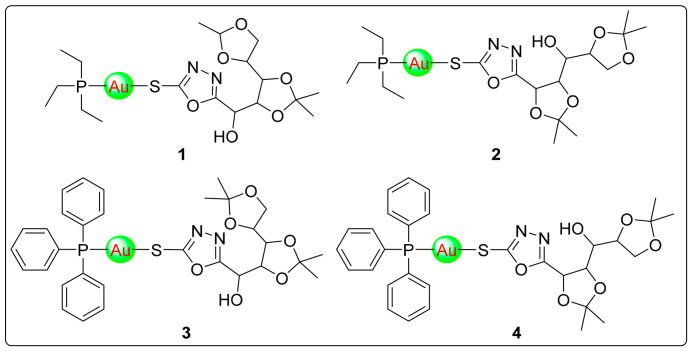
Structure of gold(I) complexes **1**–**4**.

A series of 4 different gold(I) complexes with *δ*-d-gluconolactone ligand (**1**–**4**) has been reported by Espinosa et al., and cytotoxic effects of these complexes were evaluated on both tumor (4T1, B16-F10) and non-tumor (BHK-21) cell lines (Figure 4).

Among all, complex **1** was found to be most active against B16-F10 melanoma cells with an IC_50_ value of 2 µM, while complex **2** was found to be most potent against the 4T1 breast cancer cell lines with an IC_50_ value of 2.6 µM. In this study, cisplatin was used as a standard with observed IC_50_ values of 6.4 µM and 6.2 µM against B16-F10 and 4T1 cell lines, respectively. This study indicates that these novel gluconolactone-based gold complexes are more potent than the standard cisplatin (Table 1) [57].

Even though no mechanistic studies were performed and SAR was discussed in the actual work, correlation of ligands with calculated IC_50_ values suggests that substitution on the phosphine plays a crucial role in governing the cytotoxic effects. The enhanced antitumor activity of gold complex with PEt_3_ ligand may be directly linked with its electronic and steric properties; however, further research is required to confirm this speculation.

**Figure 5:**
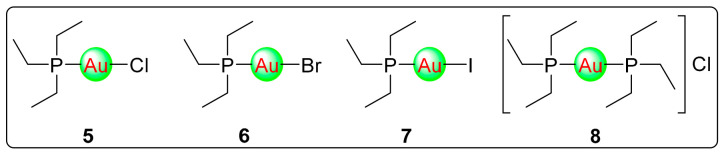
Structures of mono-phosphine and di-phosphine-based Au(I) complexes.

Cirri et al. reported auranofin-inspired new series of Au(I) and Ag(I) complexes and evaluated their anticancer potential against leukemia cancer cell lines, along with proteosome inhibitory properties. Since we are compiling Au(I) complexes with anticancer potential, only gold complexes (**5**–**8**) are discussed here (Figure 5).

Complex **8** was found to be highly active with an IC_50_ value of 0.19 µM against CCRF-CEM and 0.23 µM against CEM/ADR5000, while complex **6** exhibited the second highest activity (Table 2). In proteosome inhibition analysis, complex **6** has shown the most potent proteosome inhibitory effects, with an IC_50_ value of 1.4 μM against the chymotrypsin-like and 1.26 μM against the trypsin-like. The strong inhibition of these proteolytically important activities may disrupt protein degradation and cellular homeostasis, thereby contributing to cancer cell death [58].

Preliminary SAR investigation of complexes **5**–**8** indicates that substitution of halide ligands like Cl, Br, and I exhibits minor changes in cytotoxic activity. In addition, halide ligands exert measurable effects on proteasome inhibition, with bromide ligands being the most active.

**Figure 6:**
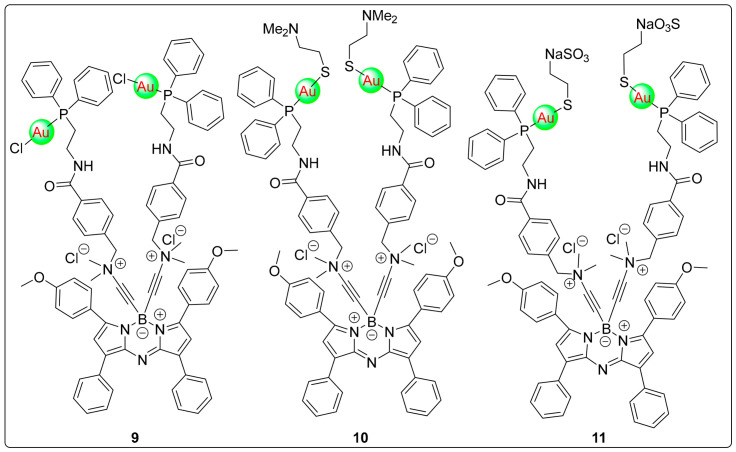
Structures of the sulfur-based gold complexes **9**–**11**.

Lescure et al. studied the antiproliferative activity of sulfur-based gold complexes **9**–**11** against human and murine cancer cell lines, i.e., 4T1, MDA-MB-231, CT26, and SW480 (Figure 6). Along with the cytotoxicity assay, thioredoxin reductase (TrxRs) activity was also performed.

Complexes showed the most notable activity against the Human Mammary Epithelial Cell (HMEC) line, ranging from 3.4 to 5.6 μM, followed by the CT26 murine colon carcinoma cell line (4.1–10.1 μM) (Table 3). Complex **9** was found to be the most active against the 4T1 cancer cell line with an IC_50_ of 4.3 μM [59].

Biological evaluation of complex **9**–**11** provides valuable insight into the ligand modification and its effects on biological activity. Replacement of the Au-Cl bond (**9**) with Au-S thiolate ligands in Complex **10**,**11** improves the cellular uptake but does not enhance the potency. These findings indicate that the potency of these complexes depends on the reactivity of the gold ion rather than the accumulation of the complex inside the cancer cell.

**Figure 7:**
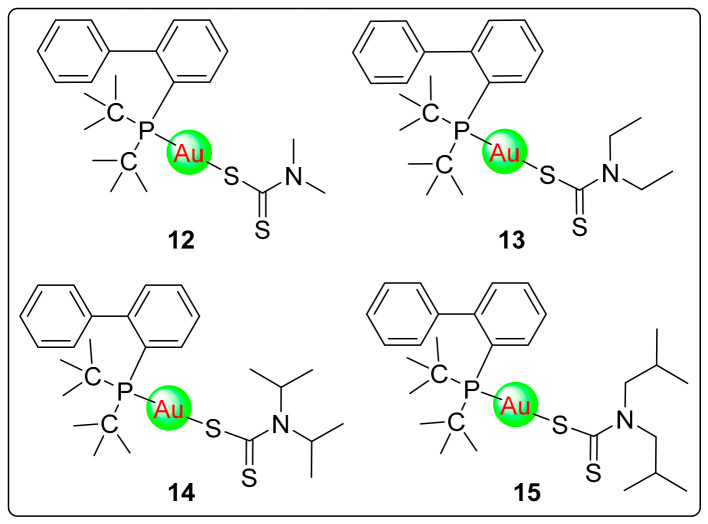
Chemical structures of complexes **12**–**15**.

Le et al. have reported four gold(I) phosphane complexes containing dialkyl dithiocarbamate ligands and evaluated their anticancer properties against highly aggressive ovarian cancer cell lines (Figure 7). The complexes have exhibited potent anticancer activity in both cisplatin-sensitive (A2780) and cisplatin-resistant (A2780cis) ovarian cancer cells.

Among the series, complex **13**, with diethyl dithiocarbamate ligands, has shown the highest anticancer activity against the A2780 cell line, with an IC_50_ value of 0.023 μM against A2780 and 0.038 μM against A2780cis (Table 4). Further, mechanistic evaluation revealed that complex **13** induced oxidative stress and ER stress-mediated p53-independent apoptosis and G2/M cell-cycle arrest. Notably, the improved activity of these complexes against cisplatin-resistant cells highlights their significance in overcoming platinum resistance, a major challenge in ovarian cancer therapy [60].

SAR analysis suggests that modification in the dialkyldithiocarbamate ligands affects the cytotoxic potency multiple times. In contrast, dimethyl **12**, diisopropyl **14**, and diisobutyl **15** analogs displayed comparatively lower activity than diethyl **13**. These findings indicate that the nature of *N*-substituents on the dithiocarbamate ligands plays a key role in improving the cytotoxicity profile of the complexes.

**Figure 8:**
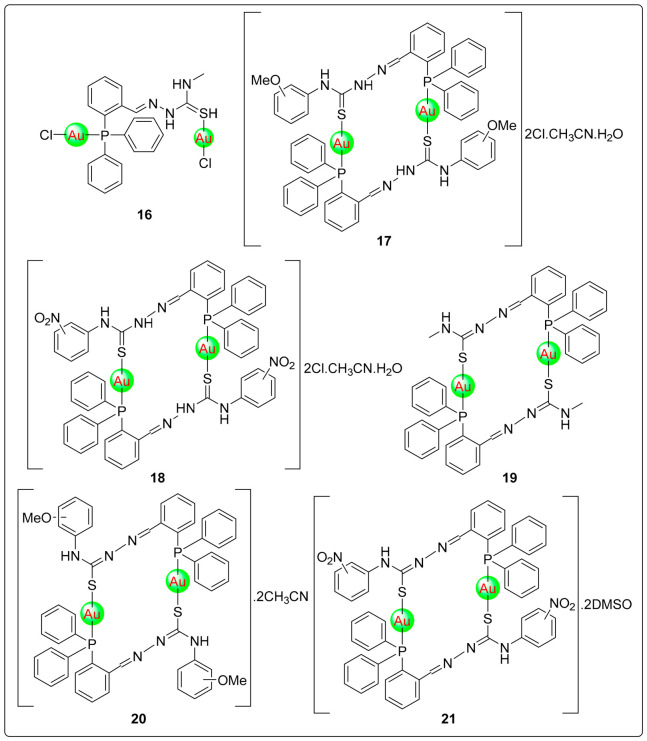
Chemical structures of the gold(I) complexes **16**–**21**.

Gonzalez-Barcia et al. reported the synthesis and anticancer effects of di-nuclear phosphine-thiosemicarbazone gold(I) complex (Figure 8). The cytotoxicity of gold(I) complexes **16**–**21** was tested against human tumor cell lines, including lung carcinoma NCI-H460, breast adenocarcinoma MCF-7, and human cervical adenocarcinoma HeLa 229 cancer cell lines, using cisplatin as a standard. Complex **16** appeared as the most active against the Hela 229 cell line with an IC_50_ value of 1.79 μM, followed by NCI-H460 and MCF-7 cell lines (Table 5). Complexes **16** and **19** induced apoptosis in HeLa 229 cells, as shown by the TUNEL assay. The inhibition of TrxR activity by complexes **16** and **19** was also investigated using the DTNB reduction assay. Overall, these assays revealed the potential anticancer effects of the gold(I) complexes [61].

SAR analysis disclosed that the nature and size of the ligand substituents significantly affect the anticancer potential of Au-complexes. Complexes **16** and **19** with an *N*-methyl substituent demonstrate the highest potency against all the tested cell lines, suggesting that a small alkyl group is favorable for activity. In addition, neutral complex **20** exhibited enhanced activity compared to its cationic derivative **17**, indicating that charge also imparts a crucial role in altering biological activity.

### 3.2. Phosphine-Nitrogen Donor Complexes

**Figure 9:**
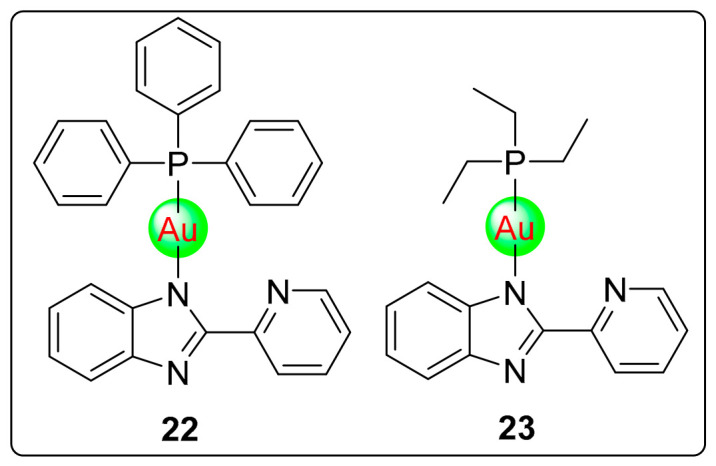
Structures of gold complexes **22** and **23**.

Rouco et al. designed and synthesized benzimidazole Au(I) complexes containing triphenylphosphine **22** and triethylphosphine **23** co-ligands (Figure 9) [50].

Complexes **22** and **23** were studied for their cytotoxic effect against the neuroblastoma cell line (SH-SY5Y). The IC_50_ values were found to be 2.7 and 1.6 µM for complexes **22** and **23**, respectively (Table 6). Complex **23**, containing triethyl phosphine co-ligand, was found to be more cytotoxic against the tested cells than complex **22** having triphenylphosphine. These complexes induced pro-apoptotic effects via caspase-dependent and caspase-independent pathways. The anticancer property of these gold(I) complexes is primarily due to TrxR inhibition-mediated oxidative stress [50].

The present investigation reveals that the higher potency of complex **23**, containing triethylphosphine as a co-ligand, might be due to the steric properties of phosphine ligands. This approach can be applied to optimize the therapeutic efficacy of new gold(I) complexes.

**Figure 10:**
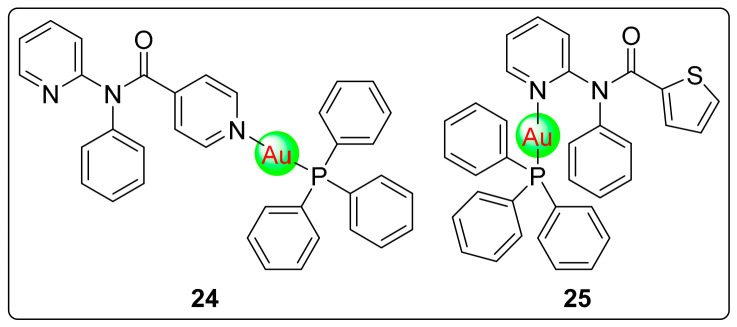
Structures of gold complexes **24** and **25**.

Mármol et al. synthesized anilinopyridine-derived Au(I) complexes with phosphine co-ligand (Figure 10). These mononuclear gold complexes were evaluated for their anticancer properties on the colorectal adenocarcinoma Caco-2 cancer cell line, MCF-7, differentiated Caco-2, and MDA-231 tumor cell lines. Additionally, they measured caspase-8 and caspase-3 pathways, and changes in mitochondrial membrane potential.

Complex **24** exhibited the most potent effects with the IC_50_ value of 2.23 µM against the Caco-2 cell line and 0.46 µM against the MCF-7 cell line, whereas complex **25** showed the most potent activity against the MDA-231 cell line with an IC_50_ value of 0.27 µM (Table 7). Further investigations revealed that complex **24** inhibited thioredoxin reductase (TrxR) and the 20S proteasome. Mechanistic studies show that complex **24** elevated the intracellular ROS level, disrupted mitochondrial membrane potential, and activated caspase-8 and caspase-3 pathways [42].

**Figure 11:**
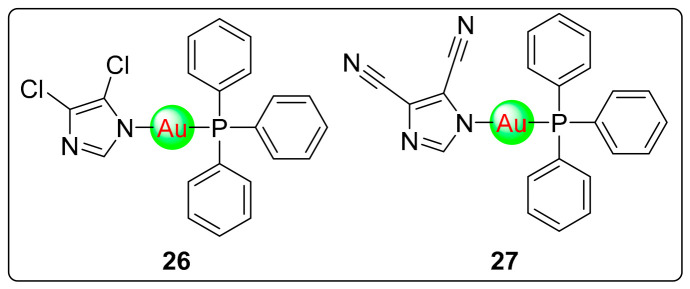
Representation of the gold(I) complexes **26** and **27**.

Gambini et al. conducted a study in which they examined seven gold(I) azolate/phosphane complexes (Figure 11). These complexes were assessed for their therapeutic efficacy against human MDA-MB-231 cells, human mammary epithelial HMLE cells overexpressing FoxQ1, and murine A17 breast cancer cell lines.

Complexes **26** and **27** demonstrated significant activity and were selected for an in vivo study in A17 tumors. These complexes exhibited greater therapeutic efficacy compared to cisplatin while being less nephrotoxic, making them promising candidates for further investigation. Complex **26** displayed better activity against A17 and HMLE/FoxQ1 cells with the IC_50_ values of 11.38 and 7.41 µM, respectively (Table 8) [62].

SAR investigation of these seven gold complexes revealed that lipophilicity is a key determinant for anticancer therapeutic efficacy. Complexes **26** and **27**, with a highly lipophilic triphenylphosphine co-ligand, demonstrate the highest cytotoxicity against all the tested cell lines. On the contrary, the introduction of hydrophilic groups such as carboxylic acid and alcohol decreases the therapeutic values, most likely due to a reduced cellular uptake. Ultimately, the results infer that the presence of the Au-PPh_3_ pharmacophore is crucial for the anticancer potential of these gold(I) complexes.

**Figure 12:**
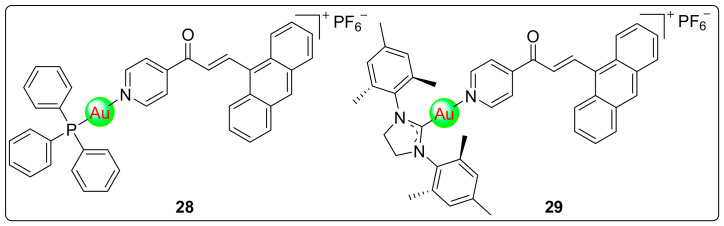
Chemical structure of complexes **28** and **29**.

Gonzalez et al. synthesized pyridyl chalcone gold(I) conjugate complexes **28** and **29** (Figure 12) and evaluated them for antitumor activity against three cancer cell lines, including human colon, melanoma, and breast cancer cell lines, with low micromolar IC_50_ values (Table 9).

The HT29, HCT-116wt, HCT116-p53, and MCF-7Topo cell lines were used for the study. Results revealed that complex **28** was prominent in the HT29 cell line, with an IC_50_ of 2.1 ± 0.1 µM, followed by the HCT116-p53 cell line with an IC_50_ of 3.6 ± 0.1 µM (Table 9). Complex **29** showed the most prominent activity against the 518A2 cell line, with the IC_50_ value of 1.1 ± 0.1 μM, followed by the activity against the DLD-1 cell line, having an IC_50_ value of 3.6 µM [63].

### 3.3. Phosphine-Carbon Donor Complexes

Organometallic compounds containing gold in their +1-oxidation state and a carbon-based ligand are known as carbon-derived gold(I) complexes. Because of their prospective uses in catalysis, material science, and medicinal chemistry, these complexes have received a lot of interest [64,65]. These compounds have unique properties, such as high surface area, biocompatibility, and tunable surface chemistry, which make them attractive for biomedical applications.

**Figure 13:**
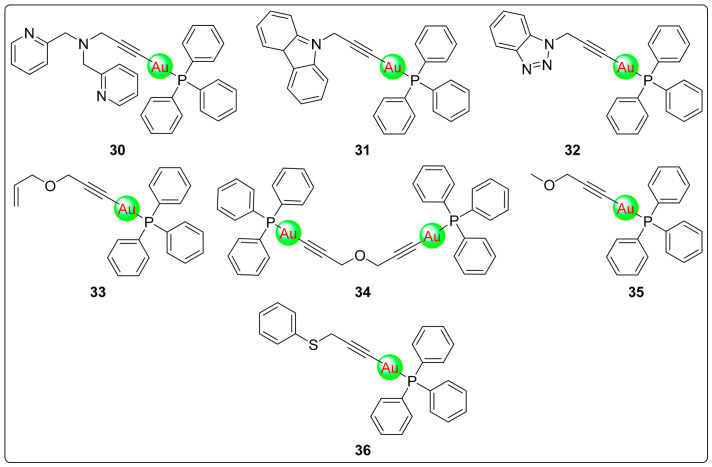
Structural representation of complexes **30**–**36**.

Johnson et al. synthesized propargyl gold(I) complexes (**30**–**36**) functionalized with different heteroatoms (N, O, or S) (Figure 13). Synthesized complexes were examined for their anti-malignant activity against A549 cells by incubating them for 24 h (Table 10). Among the synthesized complexes, complex **36** was found to be most active with an IC_50_ value of 9.11 ± 1.93 μM against the A549 Cell line [66].

SAR analysis of the propargyl gold(I) complexes **30**–**36** revealed that the anticancer activity is strongly influenced by the nature of the terminal propargyl substituent. The sulfur-containing complex **36** and the allyloxy-substituted complex **33** exhibited the highest cytotoxic activities, suggesting that these functionalities are favorable for antiproliferative effects. Complexes bearing nitrogen-containing heterocyclic substituents displayed moderate activity, whereas the carbazole ligand-containing complex **31** showed a noticeable decrease in potency, which may be attributed to the bulky and rigid nature of the carbazole scaffold. The binuclear complex **34** retained good cytotoxic activity; however, its potency remained lower than that of complex 36, indicating that the chemical nature of the terminal substituent exerts a greater influence on biological activity than the presence of an additional gold(I) center. Overall, sulfur-containing and allyloxy-functionalized ligands appear to be the most favorable structural features within this series.

**Figure 14:**
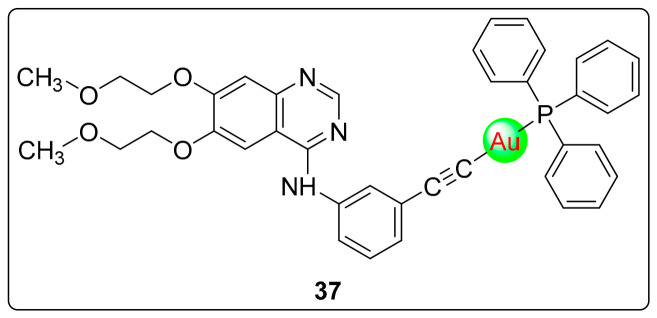
Structure of the gold complex **37**.

Ortega et al. prepared a gold(I)–erlotinib complex **37** with triphenylphosphine co-ligand against a well-known target EGFR (Figure 14), as the erlotinib inhibits EGFR tyrosine kinase and induces cell cycle arrest at G_1_-phase. The synthesized complex was examined for its therapeutic potential against human breast cancer, i.e., MDA-MBA-231, HT-29, and MCF-7 colon cancer cell lines (Table 11).

The prepared gold complex was found to be more potent than erlotinib in the studied cancer cell lines. Complex **37**, with an IC_50_ value of 1.64 µM, was found to be 41-fold more cytotoxic than erlotinib, having an IC_50_ value of 68.11 µM against MDA-MB-231 cancer cells. Complex **37** also showed promising activity against HT-29 and MCF-7 cancer cell lines with IC_50_ values of 3.90 and 2.26 µM, respectively. Further, mechanistic studies revealed that complex **37** induces intracellular ROS production, mitochondrial dysfunction, and apoptosis. Cell cycle assay exhibits arrest primarily in the G2/M and S Phase [67].

SAR investigation revealed that conjugation of phosphine gold(I) scaffold with erlotinib pharmacophore is a favorable structural modification strategy as it improved the antiproliferative activity of the new complex by many folds compared to the parent drug alone. These research outcomes highlight that API conjugation with a gold(I) scaffold is a unique and effective ligand design strategy to develop novel anticancer gold(I) complexes.

**Figure 15:**
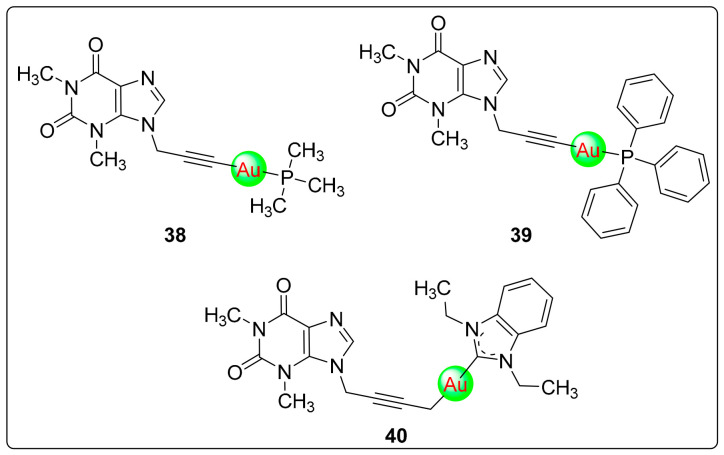
Structures of xanthine-derived alkynyl gold(I) complexes **38**–**40**.

Zhang et al. designed and synthesized xanthine-containing alkynyl phosphane gold(I) complexes **38**–**40** (Figure 15), and evaluated their effects on various biological parameters, including anti-metastatic, selective proliferation inhibitory, aneuploidy in HCT-116 colon carcinoma cells, and anti-angiogenic effect. In this study, the potential anti-metastatic effect was investigated using highly migratory MDA-MB-231 cells through the wound healing assay.

Highest cytotoxicity was achieved after 96 h of treatment with IC_50_ values in the range of 6.65–25.8 µM for **38** and 2.93–13.9 µM for **39**. Targeting low-aneuploidy-tolerance colorectal tumors may be possible using comparable complexes **38** with the most prominent action against cancer cell lines. Drug combinations with these many biological effects can significantly lower the potential for systemic toxicity [68].

SAR analysis confirms that the gold(I) center and ligand coordination play a crucial role in determining therapeutic efficacy. The xanthine ligand itself is inactive (IC_50_ > 100 μM); the corresponding gold(I) complex **38** showcased remarkable activity, which confirms that the Au(I)-phosphane scaffold is essential for biological responses. Additionally, complex **38** showcased strong TrxR inhibition with an IC_50_ of 0.014 μM ± 0.002, which is 10 times that of complex **39** (IC_50_ value of 0.110 μM ± 0.028), indicating that the smaller size of the phosphane ligand might be resulting in higher activity. In contrast, complex **40** was found inactive, which the authors assumed was due to compromised solubility, suggesting that physicochemical parameters have a strong influence on therapeutic efficacy.

**Figure 16:**
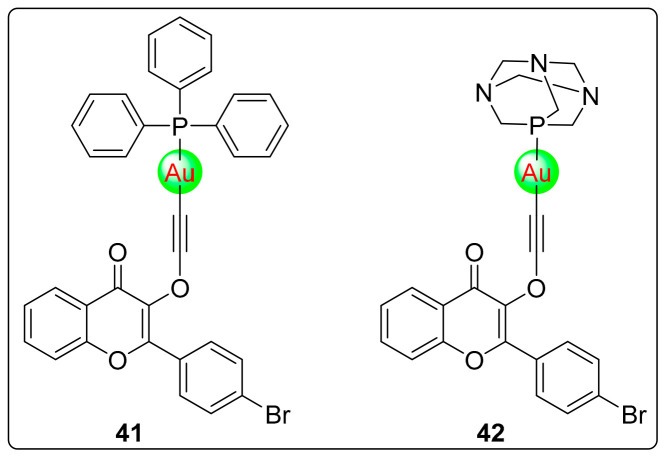
Structures of flavones and their gold complexes **41**–**42**.

Ines Marmol et al. worked on the 3-hydroxyflavone-derived alkynyl gold(I) phosphane complexes (Figure 16) and found them to be effective against Caco-2/TC7, MCF-7, and hepatocellular carcinoma HepG2 cells (Table 12). Rationale to select flavone-based ligand was to design molecules that can act via multiple targets, as the flavone moiety acts as an anticancer agent by targeting multiple pathways.

Complex **41** has shown prominent activity against the Caco-2/TC7 cell line with an IC_50_ value of 1.52 µM, whereas complex **42** has shown good activity, having an IC_50_ value of 2.33 µM. Complexes **41** and **42** also showed good activity against the HepG2 cell line, having the IC_50_ values of 3.38 and 5.88 µM compared to other cell lines (Table 12) [69].

SAR investigation revealed that incorporating bioactive molecules such as 3-hydroxyflavone as a ligand for the gold(I) complex is beneficial for anticancer activity. However, substitution of the phosphine ligand with a polar group, complex **42**, leads to a decrease in biological response, indicating that ligand lipophilicity and coordination environment also determine the therapeutic outcomes.

**Figure 17:**
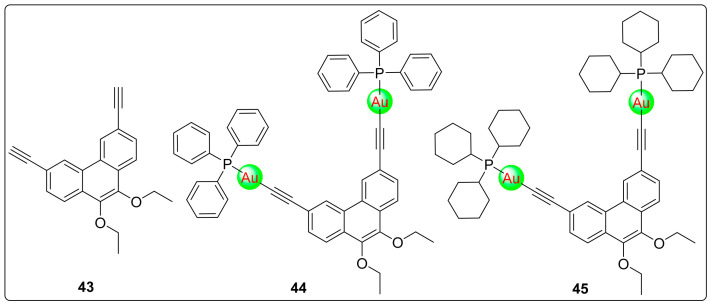
Chemical structures of ligand **43** along with gold complexes **44** and **45**.

Alsaeedi et al. synthesized ligand **43** from phenanthrenyl and prepared its binuclear gold complexes **44** and **45** (Figure 17). The anticancer activity of the synthesized compounds and ligands was evaluated against several cancer cell lines.

Complexes **43**, **44**, and **45** were studied against the PC-3, HEPG-2, MOLT-4, and MCF-7 cell lines. Results revealed that complex **45** was found to be the most efficient against the MCF-7 cell line, having the IC_50_ value of 18.63 µM, and was also observed to be the most stable as compared to the other complexes (Table 13). Complex **44** has good activity against all tested cell lines, having IC_50_ values in the range of 22.58–27.46 µM, and in that complex, **45** has shown the most potent activity in all the complexes [70].

The above investigation revealed that coordination of the ligand to a gold(I)–phosphine scaffold drastically improves the biological activity, as complexes **44** and **45** exhibited nearly 10-fold higher cytotoxicity than the parent ligand **43**. Furthermore, replacement of the triphenylphosphine ligand with tricyclohexylphosphine had only a minor effect on the anticancer activity, indicating that the presence of the gold(I)–phosphine scaffold is more critical for biological potency than the specific nature of the phosphine ligand.

**Figure 18:**
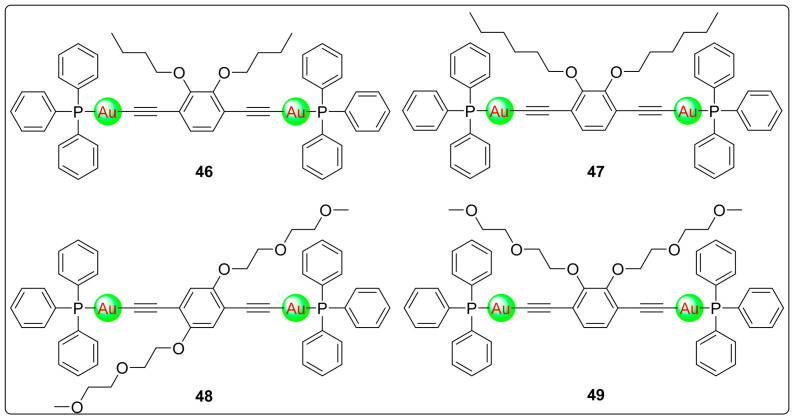
Chemical structures of complexes **46**–**49**.

Chen et al. synthesize a series of novel alkynyl (phosphine) digold(I) complexes **46**–**49** (Figure 18). These complexes feature two groups inserted into the 1,4-diethenylbenzene bridge at separate positions (2,5- and 2,3-positions).

The antitumor activities of several alkyl and oligo (ethylene glycol) methyl ether substituted complexes were evaluated in vitro against tumor and normal cell lines (Table 14). Among them, complexes **46** (1.74 ± 0.04 µM) and **47** (1.93 ± 0.08 µM) demonstrated higher activity than cisplatin (3.16 ± 0.36 µM) against HeLa cell lines. Notably, complex **47** exhibited broad-spectrum antitumor activity against multiple cancer cell lines. Complex **48** (3.06 ± 0.21 µM), which featured oligo (ethylene glycol) methyl ether substitution, exhibited similar activity to cisplatin (3.01 ± 2.91 µM) against the PC12 cell line. Furthermore, complex 48 also showed enhanced activity against A549 cells (IC_50_ = 0.69 ± 0.19 µM) compared with cisplatin (IC_50_ = 1.12 ± 0.12 µM). Interestingly, complex **49** (0.02 ± 0.01 µM) displayed significantly higher activity than cisplatin (1.12 ± 0.12 µM) against the A549 cell line, approximately 56-fold more active. Furthermore, complex **49** showed lower cytotoxicity towards three normal cell lines compared to cisplatin. The remarkable antitumor activity of complex **49** against the A549 cell line, coupled with its lower toxicity towards normal cells, may be associated with improved hydrophilicity imparted by the oligo(ethylene glycol) methyl ether substituents, which could contribute to a favorable hydrophilic–lipophilic balance. These observations highlight complex **49** represents a promising lead candidate for further development as an antitumor agent [71].

Biological studies on complex **46**–**49** unveil that both the substituent position and the nature of the side chain affect the cytotoxic potential of gold(I) complexes. In contrast, derivatives with 2,3-substitution (complexes **47** and **49**) exhibited increased potency compared to 2,5-substituted derivatives (complexes **46** and **48**). Additionally, the substitution of an oligo(ethylene glycol) substituent significantly improves the potency against A549 (complex **49**). These outcomes indicate that substitution pattern and physicochemical properties like hydrophilic–lipophilic balance govern the anticancer properties of alkynyl digold(I) complexes.

## 4. N-Heterocyclic Carbene (NHC)-Based Gold(I) Complexes

Gold complexes can be formed by coordinating nitrogen-containing heterocyclic fragments with gold atoms. For example, pyridine, a heterocyclic compound, can coordinate with gold through its lone pair of electrons on the nitrogen atom [72,73,74]. Similarly, imidazole, pyrrole, and triazole heterocycles with the presence of lone pairs of electrons on their nitrogen atoms can also coordinate with gold ions. Such gold complexes, involving nitrogen-containing heterocyclic fragments, have been demonstrated for a wide range of uses, including catalysis, sensing, and medicinal chemistry applications [73].

### 4.1. Mono/Di Carbene Gold(I) Complexes

**Figure 19:**
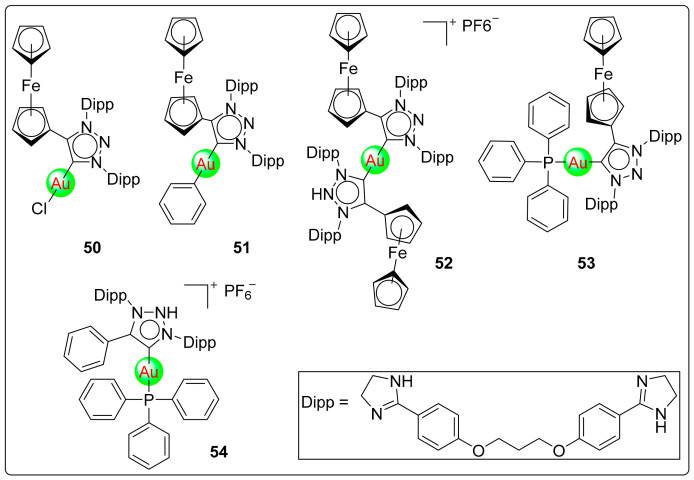
Chemical structure of gold(I) triphenylphosphine triazolylidene complexes **50**–**54**.

Aucamp et al. conducted a study focusing on the synthesis of 1,2,3-triazol-5-ylidene (trz) complexes of gold(I) with a ferrocenyl substituent on the C4 position of the trz ring **50**–**54** (Figure 19).

The anticancer potential of complex **53** against A549 and H1975 was evaluated (Table 15). To establish a non-cancer model, they utilized the human embryonic kidney cell line (HEK-293). The IC_50_ values demonstrate the selectivity and activity of compound **53** for cancer cells. The complex **53** exhibited the most favorable action on the H1975 cell line with an IC_50_ value of 0.23 µM [75].

**Figure 20:**
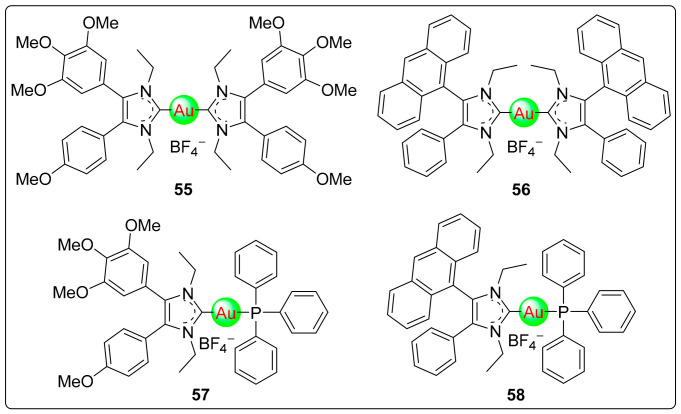
Structural representation of complexes **55**–**58**.

Bar et al. investigated a series of imidazol-2-ylidene gold(I) complexes **55**–**58** (Figure 20). These complexes were subjected to anticancer evaluation against HCT-116wt, HCT-116P, 518A2 melanoma, HeLa, KB-V1 cervical carcinomas, and human adult dermal fibroblast cells HDFa and also evaluated for intracellular localization.

Among these complexes, complex **55** was found to be the most potent, having IC_50_ values in the range of 0.05–4.6 µM against all tested cancer cell lines (Table 16). Complex **55** showed the maximum cytotoxicity against the HT-116P cancer cell line with an IC_50_ value of 0.05 µM. Additionally, complex **56** also revealed significant cytotoxicity against HT-116P and HCT-116 cancer cells with IC_50_ values of 0.2 and 0.3 µM, respectively. Complex **57** showed promising anticancer activity against the HT-116P and KB-V1 cancer cells with an IC_50_ value of 0.6 µM for each cell line. Complex **58** showed the maximum cytotoxicity against the HT-116P cell line, with an IC_50_ value of 0.4 µM [76].

SAR study reveals the superiority of bis-NHC gold(I) complexes over the corresponding NHC-phosphine complexes. Particularly, the NHC ligand with a methoxy substituent was more potent than the bulkier anthracene-substituted NHC ligand.

**Figure 21:**
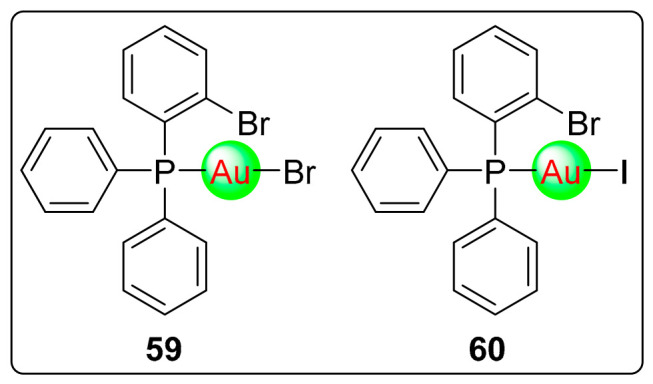
Structures of neutral gold(I) complexes **59** and **60**.

Reddy et al. reported mononuclear gold(I) complexes incorporating the 2-BrC_6_F_4_PPh_2_ ligand (Figure 21). These complexes were investigated for anticancer activity against five different human tumor cell lines, namely prostate (PC3), glioblastoma (U87MG), cervical (HeLa), fibrosarcoma (HT1080), and ovarian (SKOV-3). Additionally, they also evaluated the effects of these complexes on a normal human embryonic kidney cell line (HEK-293 T). Two of the most potent complexes are discussed below.

Among all tested complexes, complex **60** was found to be the most potent complex of the series, having IC_50_ values in the range of 1.96–12.3 µM against all tested cancer cell lines and TrxR inhibition with EC_50_ = 0.11 μM. (Table 17). Complex **59** was found to be the most potent against the U87MG cell line and has an IC_50_ value of 7.31 µM [77].

SAR analysis of mononuclear gold(I) complexes revealed that both the phosphine ligand and coordinated halide co-ligand have influence on cytotoxic effects and TrxR inhibition. Cytotoxicity increased in the order I > Br > Cl, with the iodido complex **60** showcasing the highest cytotoxicity and TrxR inhibition. This pattern indicates heavier halide offers higher biological responses.

**Figure 22:**
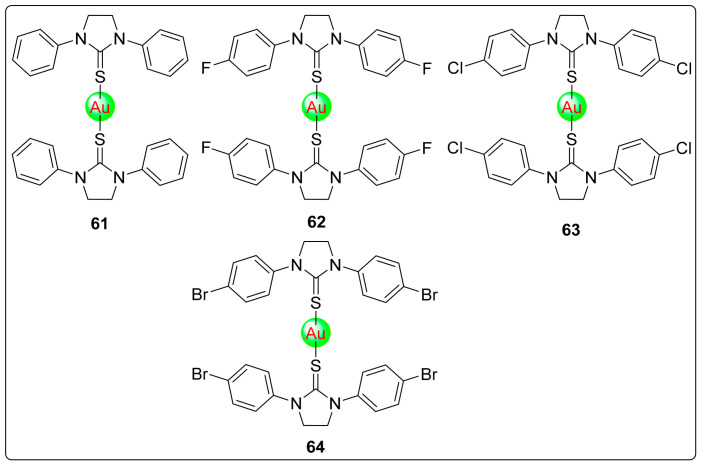
Structure of synthesized gold complexes **61**–**64**.

Yu et al. studied the therapeutic potential of newly synthesized Au(I) complexes containing *N*, *N*-disubstituted cyclic thiourea ligands towards HeLa, MCF-7, and SGC-7901 cancer cell lines (Figure 22). All the complexes exhibited excellent anticancer activity, having low IC_50_ values toward the tested cancer cells (Table 18).

Complex **63** exhibited noteworthy cytotoxicity against Hela cells, as evidenced by an IC_50_ value of 8.06 µM. The HeLa cell line was found to be the most sensitive among all tested cell lines for all complexes, having IC_50_ values in the range of 8.06–19.27 µM. Complex **62** was found to be the most effective against the SGC-7901 cell line and has an IC_50_ value of 18.52 µM, whereas complex **63** has an IC_50_ value of 24.23 µM [46].

Biological investigation indicates that the cytotoxicity of these complexes is greatly influenced by the halogen substituent on the para position of the aromatic ring. The p-chloro-substituted complex **63** exhibited the most potent cytotoxicity, particularly against HeLa cell lines; in contrast, fluoro-substituted complex **62** and bromo-substituted complex **64** were less active. These findings infer that the size of halogen alone does not affect the activity; an optimal balance of lipophilicity, steric properties, and electron-withdrawing effects favors the potency.

**Figure 23:**
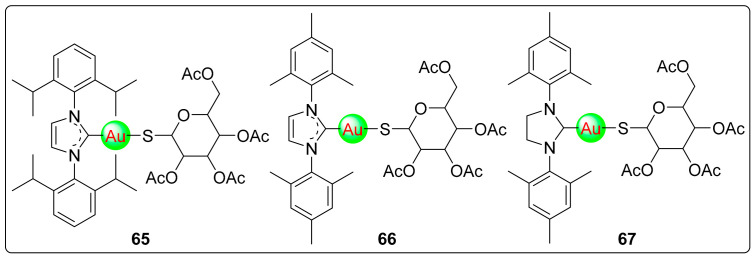
Structures of gold–NHC thiolato complexes **65**–**67**.

Filho et al. synthesized a series of thiosugar-bearing gold(I) complexes **65**–**67** (Figure 23) by combining four different *N*-heterocyclic carbenes (NHC) ligands.

Complexes **65**, **66**, and **67** have strong cytotoxic activities against lung, colon, and breast cancer with IC_50_ values in the submicromolar range (Table 19). Biological assessments revealed that complexes **65**, **66**, and **67** showed selectivity against the MDA-MB-231 cell, having IC_50_ values in the range of 0.7–1.0 µM (Table 19). All complexes also showed significant anticancer activity against A-549 cancer cells with IC_50_ values in the range of 2.2–3.0 µM. Complex **66** was found as the most active, having IC_50_ values of 0.7 µM against the MDA-MB-231 cell line, and 2.2 µM and 1.0 µM against the HT-29 and A-549 cell lines, respectively [78].

The comparative biological evaluation of complexes **65**–**67** suggests that peracetyl thioglucose is an essential pharmacophore. Apart from improving lipophilicity and cellular uptake, the thioglucose scaffold may facilitate accumulation in cancer cells by exploiting their increased glucose uptake and metabolism, a well-known phenomenon called the Warburg effect. In addition, substitution of a bulkier isopropyl group in complex **65** with a simple methyl group in complex **66** imparts minor improvement in activity, suggesting that steric hindrance around the NHC framework may diminish the biological interactions. Replacing the unsaturated benzimidazole ring of NHC framework **66** with unsaturated ring **67** moderately suppresses the activity against all the tested cell lines, indicating that a firm and π-conjugated benzimidazolyl framework is more favorable, most likely due to its electronic characteristics and stability of the gold (I) center.

### 4.2. Bis-NHC Gold(I) Complexes

**Figure 24:**
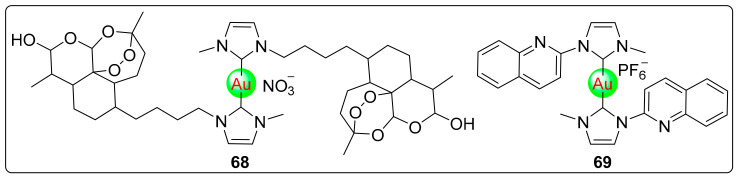
Structure of gold(I) complexes **68** and **69**.

Zhang et al. reported a biological study on a series of cationic bis NHC gold(I) complexes by incorporating dihydroartemisinin (DHA), a semi-synthetic analog of artemisinin moieties, and compared with previously reported bis (NHC) gold(I) complex **69** (Figure 24).

The synthesized gold complexes were evaluated for their anticancer potential against various tumor cell lines, including MCF-7, A549, HepG2 (hepatoblastoma), U-2 OS (bone sarcoma), T24 (bladder carcinoma), LAMA (chronic myeloid leukemia), and HL-60 (acute promyelocytic leukemia) cell lines. Among these complexes, complex **68** displayed the most promising anticancer activity, with GI_50_ values in the nanomolar range and higher selectivity compared to the standard treatments. When compared to the standard compound auranofin, which has GI_50_ values ranging from 0.474 to 4.41 µM across different cell lines, complex **68** showed superior activity and surmounted multidrug resistance in Acute Myeloid Leukemia (AML) models. Notably, Complex **68** exhibited a GI_50_ value of 0.017 µM against the HL-60 cell line, indicating strong antiproliferative activity. Table 20 provides a detailed comparison of the GI_50_ values (in µM) for complex **68**, complex **69**, auranofin, and DHA against the tested tumor cell line. Overall, complex **68** demonstrated significant growth-inhibitory activity across a broad range of cancer cell lines. Among the evaluated cell lines, complex **68** exhibited the highest activity against HL-60 cells, with a GI_50_ value of 0.017 µM. These findings emphasized DHA-substituted bisNHC ligand is a promising anticancer scaffold to develop potent anticancer gold(I) complexes [45].

### 4.3. Functionalized NHC Gold(I) Complexes

**Figure 25:**
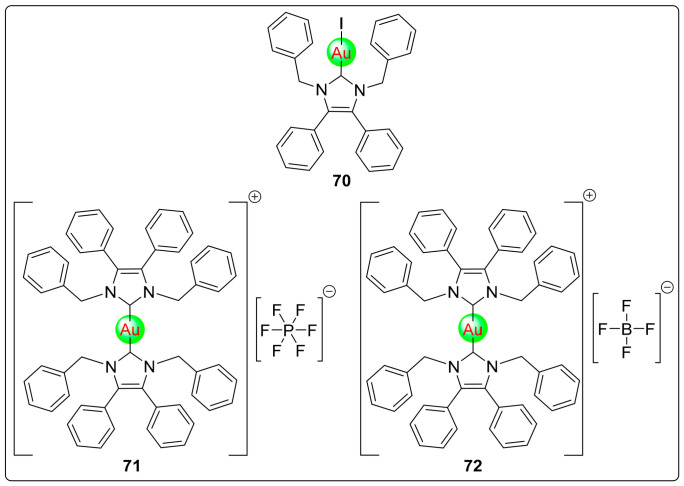
Structural representations of complexes **70**, **71**, and **72**.

Curran et al. designed, synthesized and evaluated a library of Lepidiline A, an imidazole alkaloid, and an inspired *N*-heterocyclic carbene gold(I) for their anticancer potentials (Figure 25). The anticancer activity of the compounds was evaluated against HCT-116wt (human colon carcinoma) and MCF-7^topo^ (multidrug-resistant human breast carcinoma) cell lines (Table 21).

The complex **72** has good anticancer activity against the HCT-116^wt^ cell line with an IC_50_ value of 0.29 µM, whereas complex **71** showed prominent activity against the MCF-7^topo^ cell line having IC_50_ value of 0.80 µM. All complexes revealed more sensitivity towards the HCT-116^wt^ cell line, having IC_50_ values in the range of 0.29–0.64 µM [79].

Biological assay reports of biscarbene complexes **71**–**72**, compared to monocarbene complexes **70**, highlighted that incorporating two NHC ligands notably improves antiproliferative response. The close biological responses of **71** and **72** indicate that the counterion (PF_6_^−^ vs. BF_4_^−^) has a minor influence on cytotoxicity profile.

**Figure 26:**
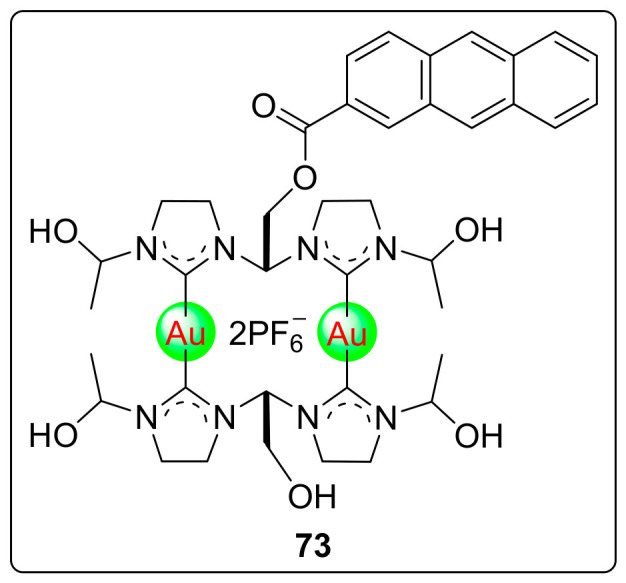
Structure of complex **73**.

Jakob et al. studied anthracene ester conjugated binuclear gold(I) bis-*N*-heterocyclic carbene complexes (Figure 26), which are studied against HeLa, MCF-7, and V79 lung fibroblast cell lines. Complex **73** has shown the most potent activity against the HeLa cell line with the IC_50_ value of 7.26 µM, followed by the MCF-7 cell line with an IC_50_ value of 7.92 µM (Table 22) [80].

Biological investigations revealed that mono-anthracene-substituted binuclear gold(I) complex **73** exhibited markedly enhanced anticancer potential compared to precursor hydroxyl-functionalized analog, highlighting that the anthracene scaffold imparts a key role in improving potency. Here, anthracene most likely improves the lipophilic character and intracellular targeting effects. Additionally, the anthracene ligand possesses fluorescent characteristics, making it an ideal choice to develop theranostic gold(I) complexes with imaging and antiproliferative properties.

**Figure 27:**
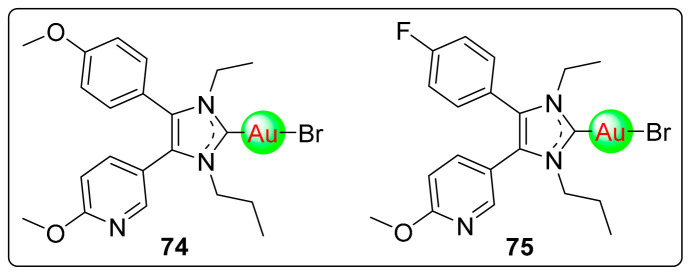
Structures of complexes **74** and **75**.

Gallati et al. conducted a study on the synthesis of 4,5-diarylimidazole-based gold(I) complexes (**74**–**75**) (Figure 27). These complexes were designed to have methyl, fluoro, or methoxy substituents at different positions on the aryl ring.

Complexes **74** and **75** were tested for their anticancer activity against MCF-7, MDA-MB-231, and HT-29 cell lines, along with cisplatin and auranofin as reference drugs. In MCF-7 cells, auranofin showed the highest activity, followed by cisplatin, complex **74**, and complex **75** (Table 23). In MDA-MB-231 cells, auranofin (2.1 ± 0.7 µM) and cisplatin (2.7 ± 0.9 µM) showed similar activity, while complex **74** (5.2 ± 0.8 µM) was less active, and complex **75** (11.0 ± 1.1 µM) showed the lowest activity. In HT-29 cells, auranofin again showed the highest activity (3.3 ± 0.0 µM), followed by complex **74** (6.3 ± 1.8 µM) and cisplatin (6.9 ± 1.8 µM), which had similar effects, while complex **75** (10.6 ± 2.8 µM) was the least active [81].

The anticancer property of complex **74** and **75** was drastically influenced by the nature of substituents on the 4-aryl ring. Methoxy derivatives **74** were found to be more active than corresponding halogen **75** derivatives, suggesting that the electron-donating effect favors cytotoxicity. Overall, methoxy substituents at the para position enhanced antitumor property, ROS generation, and improved TrxR inhibition.

**Figure 28:**
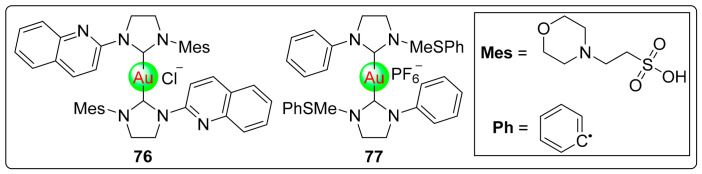
Structures of gold(I) complexes **76** and **77**.

Zhang et al.’s study focuses on the synthesis and characterization of a diverse set of 14 gold(I) complexes. These complexes included both cationic bis (NHC) and neutral mono (NHC) complexes (Figure 28).

Complex **76** exhibited significant activity against the A2780cis cell line with an IC_50_ value of 0.11 µM, followed by the HepAD38 cell line, having an IC_50_ value of 0.39 µM. Complex **77** displayed significant activity against the A2780 cell line with an IC_50_ value of 0.17 µM (Table 24) [82].

Reported findings suggested that the anticancer property of these complexes was strongly controlled by the ligand framework and the overall charge present on it. The NHC gold(I) complex with quinoline substituent **76** and thioanisole (MeSPh) substituent **77** demonstrated the highest potency against HepG2 cells, suggesting the above combination of ligands is highly favorable for cytotoxicity. In contrast, the corresponding neutral complex exhibited lower potency, emphasizing the importance of the cationic bis (NHC) framework.

**Figure 29:**
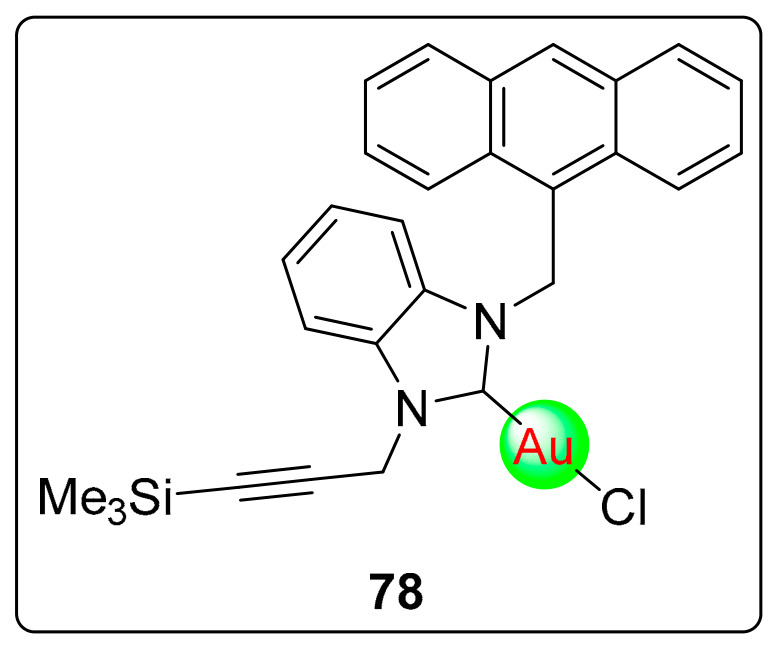
Structure of complex **78**.

Guarra et al. synthesized and characterized an Au(I) complex containing an NHC fluorescent ligand (Figure 29). In this study, the cytotoxicity of compounds was evaluated using the MTT proliferation assay across various tumor cell lines, including SW480 (colon adenocarcinoma), A549, and HepG2, as well as in the healthy cell line IMR-90 (lung fibroblasts).

The complex **78** exhibited an IC_50_ value in the micromolar range (6–20 µM) toward all the tested cell lines (Table 25). Complex **78** was the most active against the TrxR cell line, having an IC_50_ value of 6 µM, followed by the HepG2 cell line, having an IC_50_ value of 9 µM [35].

**Figure 30:**
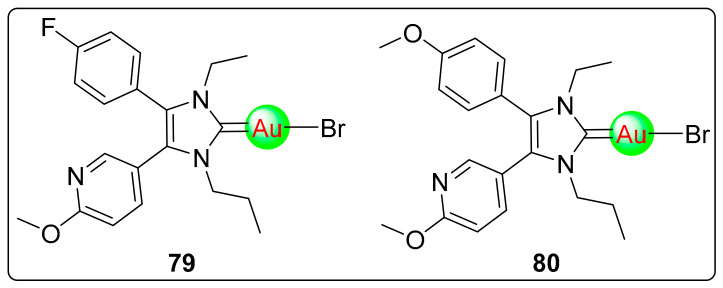
Structures of NHC gold(I) complexes **79** and **80**.

Gallati et al. studied a series of 2-methoxypyridin-5-yl-linked NHC gold(I) complexes (**79**–**80**) and evaluated them against cisplatin-resistant ovarian tumor cell lines (Figure 30). Complex **80** is most prominent against the MCF-7 cell line, having an IC_50_ value of 3.7 µM, followed by the MDA-MB-231 cell line, having an IC_50_ value of 5.2 µM (Table 26). In complex **79**, good activity is shown against the MCF-7 cell line with an IC_50_ value of 4.0 µM [81].

The methoxy substituent on complex **80** demonstrates superior activity than the corresponding fluoro complex **79**, indicating that the electron-donating effect favors biological responses; a similar trend was noticed for complexes **74** and **75**. The combination of an electron-donating group at the para position with a conjugated olefinic linker is a favorable ligand combination in Au(I)-NHC complexes.

**Figure 31:**
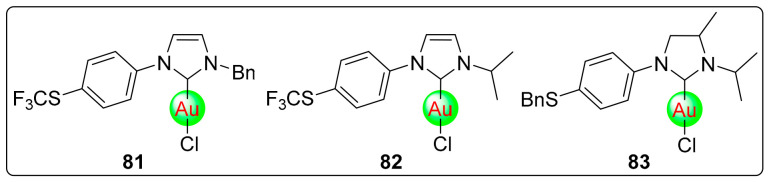
Most active complexes.

Ftouh et al. reported the synthesis of a new family of six imidazolium salts with nitrogen- or sulfur-containing side arms, as well as their corresponding neutral gold(I) complexes bearing *N*-heterocyclic carbene (NHC) ligands (Figure 31). These compounds have shown interesting biological activities in the fields of cancer, malaria, and leishmaniasis. The study specifically evaluated the in vitro antileishmanial effects on L. infantum axenic amastigotes and the cytotoxicity for the human THP1 cell line. New imidazolium salts and (NHC)AuCl complexes have been evaluated as potential therapeutic agents against leishmaniasis, while considering their cytotoxicity towards human cells [83].

All complexes **81**–**83** showed even better activity (having IC_50_ values in the ranges of 15.84–20.93 μM) in comparison to miltefosine (having an IC_50_ value of 35.02 μM). Complex **81** was found to be the potent candidate with an IC_50_ value of 20.93 μM, while complex **82** showed an IC_50_ value of 16.24 μM (Table 27).

All these complexes have outperformed the reference drug miltefosine. Replacing the benzyl substituent in complex **81** with isopropyl **82**, on the nitrogen of the imidazole ring, had mild effects on activity, making this substitution tolerable. But substituting -SF_3_ (strong electron withdrawing) group with benzylthio moiety in complex **83** diminishes the activity, indicating that the presence of a strong electron withdrawing group is acceptable.

**Figure 32:**
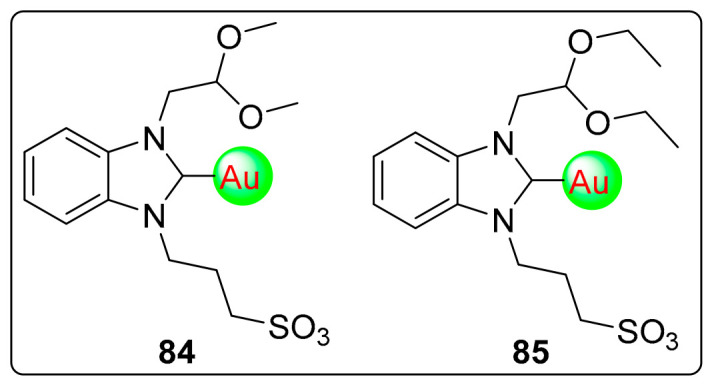
Structures of complexes **84** and **85**.

Ozdemir et al. studied two novel gold complexes of benzimidazolium salt, which contain propane sulfonate (Figure 32). Human fibroblasts (HF), adenocarcinoma (HEP3B), and neuroblastoma (SHSY5Y) cell lines were used for the study. Anticancer study results revealed that complexes **84** and **85** are most prominent against the HEP3B cell line with IC_50_ values of 9.32 and 10.51 µM, respectively (Table 28). Complex **84** also showed good cytotoxicity against the SHSY5Y cell line with an IC_50_ value of 55.16 µM [84].

The cytotoxicity data indicated that propane sulfonate side chain bearing benzimidazolylidene gold(I) complexes showcased desired anticancer potential. Comparative analysis of complex **84** and **85** disclosed that replacing the methoxy group with the ethoxy group led to a slight decrease in biological response across all tested cell lines, suggesting that small alkoxy substituents are more favorable than the larger ones. Collectively, benzimidazole-based NHC gold(I) complex bearing propane sulfonate moiety offers a promising framework for further development of anticancer gold(I) complexes.

### 4.4. Advanced NHC Architectures

**Figure 33:**
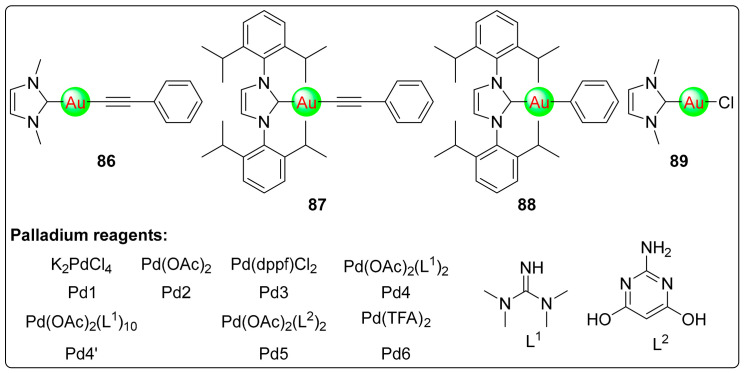
Chemical structures of complexes **86**–**89**.

Long et al. studied about living system’s activation of the bio-reactivity in metal complexes (Figure 33). This can help to improve spatiotemporal specificity and can minimize off-target bindings. Complex **86** showed a stable reaction to biological thiols. These NHC-Au(I) species show activation only inside those cells that are living, and these species can lower the activity of thioredoxin reductase, which results in lowering the effect of tumor cells [85].

The exposure of A549 cells to either complex **86** or Pd4 for 1 h did not show significant inhibition of TrxR (IC_50_ > 100 µM). However, when Pd4 was combined with complex **86**, it significantly enhanced its inhibitory activity against TrxR, resulting in an IC_50_ value of 14.8 ± 3.5 µM. This value was 7 times higher than that of complex **86** alone and even more potent than complex **89** (IC_50_ = 24.0 ± 2.3 µM). Other Pd (II) compounds, such as Pd1, Pd2, and Pd4′, which exhibited TrxR inhibitory IC_50_ values of 14.1–27.0 µM, also showed the potential to activate complex **86** similarly. The researchers then examined whether these Pd (II) molecules could trigger the cytotoxicity of gold(I) complexes. In preliminary tests with Pd4, the cytotoxicity IC_50_ of complex **86** increased from 63.3 to 17.5 µM after the addition of the Pd (II) salt. Two other non-toxic Pd (II) compounds, Pd4′ and Pd5, demonstrated slightly higher cytotoxicity with IC_50_ values of 13.1 µM. Further investigations were conducted to evaluate their cytotoxicity against human hepatocellular carcinoma HepG2, breast cancer MCF-7, and glioblastoma U87 cells. Complex **86** alone exhibited modest cytotoxicity, with IC_50_ values ranging from 52.4 to 70.6 µM. However, when Pd4′ and Pd5 were added, the cytotoxicity significantly increased to 8.5–13.1 µM and 6.4–18.5 µM, respectively (Table 29). Similarly, complex 87 showed comparable potency when activated by Pd4′ and Pd5, resulting in significant cytotoxicity improvement in HepG2 (from 84.0 to 8.5 µM) and A549 cells when co-treated with Pd4′. On the other hand, the cytotoxicity of complex 89 moderately increased after co-treatment with Pd4′, with the IC_50_ value increasing from 48.1 to 19.4 µM.

**Figure 34:**
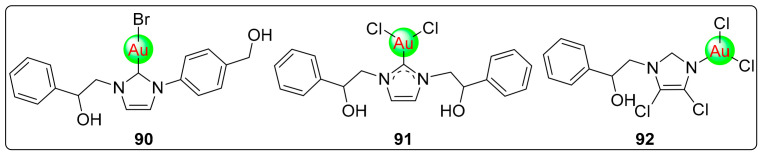
Chemical structure of complex **90**–**92**.

Iacopetta et al. studied the synthesis of the different types of antitumor properties of gold carbene complexes (Figure 34). In this study, the TUNEL assay detected apoptosis, while the caspase-Glo assay measured caspase activity. Tubulin-polymerization inhibition was assessed through turbidity changes. Human topoisomerase I and II activities were analyzed via relaxation and decatenation assays, respectively. These assays provided insights into the apoptotic effects and enzyme inhibition induced by the compounds.

Complex **92** showed the most prominent activity against the MDA-MB cell line and its IC_50_ value was 2.1 µM (Table 30), followed by activity against MCF-7 cell lines, with an IC_50_ value of 5.18 µM. In complex **90**, the IC_50_ value ranges from 18.2 to 54.9 µM. Complexes **90** and **91** were found to be the most active against the MCF-7 cell line, with the IC_50_ values of 18.2 and 9.38 µM, respectively [86].

**Figure 35:**
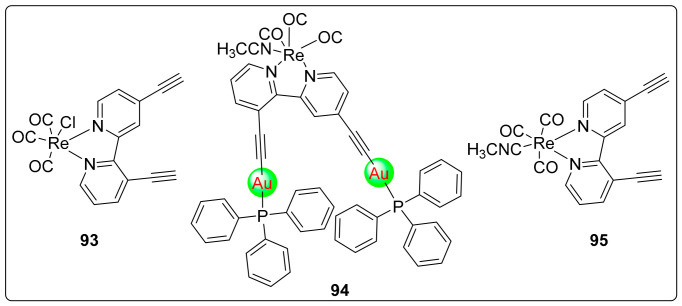
Structures of complexes **93**–**95**.

Luengo et al. studied the synthesis and characterized a series of heterobimetallic complexes of Re and Au **93**–**95** that were octahedral and linear distribution of the ligands along with the Re(I) and Au(I) centers (Figure 35).

Complexes **93**–**95** were examined for their anticancer properties. Complexes showed potent activity against A549 and HeLa cell lines after 72 h incubation compared with 24 h exposure. Among them, complex 94 exhibited the most potent activity against HeLa cells after 72 h, with an IC_50_ value of 2.35 μM (Table 31) [87].

In these complexes, the Au(I) center acts as the biologically active pharmacophore, while the Re(I) fragment serves primarily as a luminescent probe for cellular imaging and tracking. The antiproliferative activity of the complexes was evaluated against A549 and HeLa cancer cell lines using the MTT assay. Alkynyl gold derivatives exhibited delayed cytotoxicity compared with other gold complexes, requiring longer incubation times (72 h). The heterometallic complexes demonstrated enhanced selectivity toward HeLa cells, and the observed biological activity was influenced by the nature of the Au(I) ancillary ligands.

**Figure 36:**
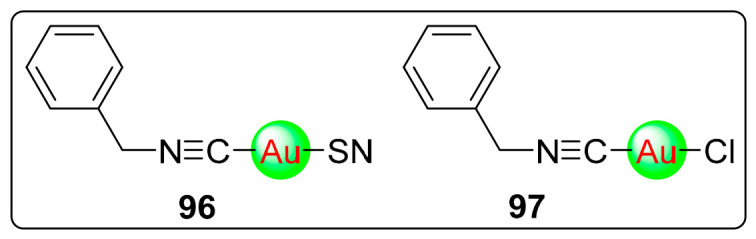
Structure of gold complexes **96** and **97**.

Faghih et al. have designed and synthesized benzyl isocyanide-based complexes **96** and **97**, and evaluated their anticancer properties against breast cancer cell lines (Figure 36).

Cytotoxic activities of these complexes were evaluated against three human cancer cell lines. Complex **96** demonstrated similar or higher cytotoxicity compared to cisplatin when tested on various cell lines. It also exhibited significant antiproliferative activity against A549, SKOV3, and MCF-7 cell lines with the IC_50_ values of 19.46, 11.76, and 13.27 μM, respectively (Table 32). On the other hand, complex **97** displayed noteworthy activity specifically against the MCF-7 cell line with an IC_50_ value of 19.14 μM [88].

These findings suggested that replacing the Au-Cl bond of complex **97** with Au-SN, complex **96** significantly enhanced the antiproliferative responses; these results indicate the importance of gold(I)-ligand in controlling biological activity.

**Figure 37:**
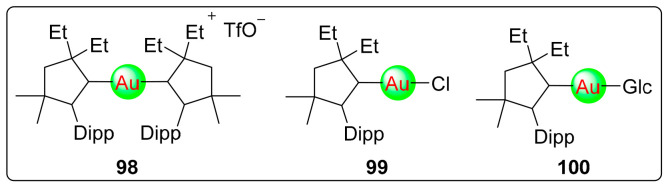
Structures of complexes **98**, **99**, and **100**.

Proetto et al. developed therapeutically active cyclic (alkyl)(amino)carbene (CAAC) gold(I) complexes **98**, **99**, and **100** (Figure 37) and evaluated them for cytotoxic effects on various cancer cell lines, including HeLa (cervical cancer), A549 (lung cancer), HT1080 (fibrosarcoma), and Caov-3 (ovarian cancer).

The complex **98** was the most active against the A549 cell line, having an IC_50_ value of 0.07 µM, followed by the HeLa and Caov-3 cell lines with the IC_50_ value of 0.3 µM for each cell line (Table 33). Complex **99** was found to be the most active against the HeLa cell line with an IC_50_ value of 0.6 µM, whereas complex **100** was found to be the most active against the Caov-3 cell line with an IC_50_ value of 1.9 µM. The significantly lower cytotoxicity of complex **98** clearly demonstrates the gold center involvement in the antitumor activity [89].

**Figure 38:**
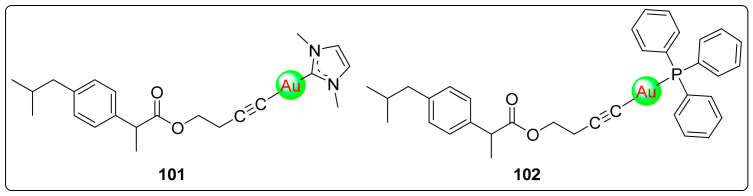
Structure of gold complexes **101**–**102**.

Tabrizi et al. synthesized ibuprofen-based gold (I) complexes (**101**–**102**) and evaluated their anticancer activity (Figure 38). The in vitro TrxR1 and TrxR2 inhibition assay used 0.2 M Na, K-phosphate buffer with NADPH and TrxR protein, monitored at 412 nm. Glutathione reductase activity was assessed at 25 °C in 0.1 M Tris–HCl (pH 8.0) with NADPH, GSSG, and monitored at 340 nm. The ROS assay measured oxidative stress in HT-29 cells using DCFH-DA dye, with fluorescence measured after treatment with complexes **101** and **102**.

The anticancer activity of all the complexes was thoroughly examined and compared with standard drugs cisplatin and auranofin (Table 34). Investigations revealed that complex **102** was more cytotoxic than complex **101** against HT-29, MCF-7, and MDA-MB-231 cancer cell lines, with the IC_50_ values 0.98, 1.25, and 1.97 µM, respectively [43].

SAR evaluation highlights that the ligand directly coordinated with the Au(I) center plays an important role. Since both complexes contain the ibuprofen ligand, the higher potency of complex **102** over complex **101** indicates that phosphine ligands are more favorable for anticancer activity.

**Figure 39:**
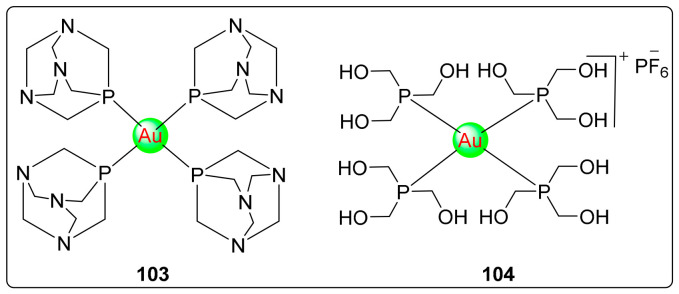
Structure of gold complexes **103** and **104**.

Ceresa et al. studied gold complexes **103** and **104**. Among these, tris(hydroxymethyl) phosphane gold(I) complex has potent anticancer activity in a wide range of solid tumors (Figure 39). This study includes proteasome inhibition activity in IGROV-1 cells and DRG neurons.

These complexes are being proposed as potential alternatives to platinum-based chemotherapeutic drugs. Complex **104** demonstrated neurotoxicity at a lower concentration compared to its IC_50_ in cancer cell lines; other complexes exhibited promising IC_50_ values in human cancer cells. Complex **104** showed the most prominent activity with an IC_50_ value of 8.3 µM (Table 35) [90].

SAR investigation suggested that the nature of the phosphine ligand drastically affects the anticancer activity. Hydroxy-functionalized ligand **104** possessed significantly higher potency than complex **103**. This enhanced activity of complex **104** may be due to its hydrophilic nature and improve cellular interactions, suggesting that hydroxyl functionalization of phosphine ligands is more favorable over amino phosphine ligands.

## 5. Clinical Translation of Gold-Derived Anticancer Complexes

Gold-derived complexes are emerging as promising anticancer agents, with increasing efforts towards their clinical translation. Among these, auranofin, an approved gold(I)-based anti-rheumatic agent, has been repurposed and evaluated in clinical trials across multiple cancer types, including ovarian cancer, leukemia, lung cancer, and glioblastoma. In addition to auranofin, other gold-based compounds such as gold sodium thiomalate have also entered clinical investigation, either as monotherapy or in combination with established therapeutic agents. Clinical studies conducted to date are primarily in Phase I and Phase II stages, focusing on safety, tolerability, and preliminary efficacy. These trials span a diverse range of malignancies, including chronic lymphocytic leukemia (CLL), non-small cell lung cancer (NSCLC), small cell lung cancer (SCLC), and glioblastoma, reflecting the broad therapeutic potential of gold-based metallodrugs. Although most candidates remain in early clinical development, the outcomes of these studies provide important insights into their feasibility and therapeutic relevance (Table 36) [91,92].

## 6. Conclusions

In conclusion, this study showcases a comprehensive survey of gold complexes showing significant anticancer potential in cell-based assays. Additionally, this new class of complex exhibits minimal side effects compared to other transition metal-based complexes such as cisplatin. Available evidence on the clinical application of gold complexes as anti-inflammatories, along with thoroughly investigated physicochemical properties of gold, has supported the exploration of gold-based anticancer agents for medical research over other metal complexes. Recently, researchers’ interest in anticancer evaluation of gold(I/III) complexes has significantly increased, resulting in the exploration of structurally diverse ligands embedded with various hetero atoms like oxygen, nitrogen, sulfur, phosphorus, and carbon. Additionally, this review summarizes gold complexes that have been explored in the past three years based on an important class of ligands called *N*-heterocyclic carbene (NHC) and their role in the development of novel Au complexes.

The majority of the complexes discussed in this review exhibited IC_50_ values in the micromolar range. Among all, complex **68**, having a bis-*N*-heterocyclic carbine donor ligand, had the lowest IC_50_ value of 0.017 μM against the HL-60 cell line, while complex **49**, having the carbon-donor ligand, had the IC_50_ value of 0.02 μM against the A459 lung cell. Furthermore, complexes **8**, **24**, **55**, **66**, **72**, **75**, **76**, **81**, and **98** were found to be the most active, having the IC_50_ values less than 1 μM against the tested cell lines, including A2780, HL-60, A549, MCF-7, MCF-10A, HT-29, and MDA-MB-231.

Overall, SAR findings suggest that the anticancer property of gold(I) complexes is governed by the framework and nature of the ligand, including electronic properties, steric bulk of substituents, lipophilicity, and coordination chemistry. Among all the ligand classes, strong donor ligands, which include phosphine, *N*-heterocyclic carbenes, sulfur donors, and carbon–carbon architecture, yielded the most potent complexes. Electronically rich aromatic scaffolds, biologically active pharmacophores, and balanced physicochemical characteristics play an essential role in improving cytotoxic potency; in contrast, steric bulk, compromised solubility, and suboptimal ligand environments usually diminish therapeutic efficacy. In addition, bis-ligand frameworks and ligands with selective intracellular accumulation properties appeared as supportive features. Furthermore, many binuclear gold(I) complexes also exhibited improved potential, highlighting that a multinuclear framework is also one of the favorable strategies for future design. However, ligand structure and physicochemical properties have a greater influence on therapeutic efficacy than merely incorporating an additional gold(I) nucleus. All of these outcomes cumulatively emphasize that ligand engineering is the fundamental factor responsible for developing next-generation anticancer gold(I) complexes.

## 7. Future Perspective

Based on the existing literature on gold(I) complexes as anticancer agents, future investigation should be grounded on sound concepts, mechanism-driven, and rationally justified in lieu of exploratory approaches. In-depth investigations and critical improvements are required to optimize and develop a more informative structural–activity relationship (SAR), which clearly reveals that alteration in ligand design, oxidation state of gold ion, and coordination geometry can alter the affinity, efficacy, and ADME profile of the complex. Concurrently, future studies should aim to unveil the mechanism of Au complexes at the molecular level and clarify the interaction of gold complexes with redox enzymes, mitochondrial pathways, and cell signaling pathways involved in cancer survival and progression. Enhancing the cancer targetability while reducing the toxicity is another key research area, and the application of molecularly engineered ligands and nanotechnology-based delivery systems can significantly improve the overall therapeutic outcomes. Application of computational tools integrated with artificial intelligence should be involved and encouraged in order to improve the accuracy of designing new Au-complexes in a short span. Ultimately, prioritizing these aspects in future study will surely assist in constructing more efficacious, tumor-specific, and clinically applicable Au-based cancer treatment.

## Figures and Tables

**Table 1:** IC_50_ (µM) values of complexes **1**–**4** and cisplatin against the cancer cell line.

Complex	B16-F10	4T1
**1**	2.0 ± 0.2	3.9 ± 0.3
**2**	3.2 ± 0.2	2.6 ± 0.2
**3**	8.3 ± 0.1	3.6 ± 0.3
**4**	7.1 ± 0.5	3.9 ± 0.1
**Cisplatin**	6.4 ± 1.0	6.2 ± 2.3

**Table 2:** IC_50_ (µM)) values of the complexes **5**–**8**.

Complex	CCRF-CEM	CEM/ADR5000
**5**	0.35 ± 0.03	0.26 ± 0.04
**6**	0.28 ± 0.06	0.27 ± 0.05
**7**	0.32 ± 0.07	0.28 ± 0.08
**8**	0.19 ± 0.06	0.23 ± 0.09
**Auranofin**	0.22 ± 0.08	0.28 ± 0.06

**Table 3:** IC_50_ values (μM) of complexes **9**, **10**, and **11** against various cell lines.

Complex	4T1	MDA-MB-231	CT26	SW480	HMEC
**9**	4.3 ± 0.5	9.7 ± 0.7	4.1 ± 0.2	4.2 ± 0.5	3.4 ± 0.9
**10**	9.9 ± 0.6	10.2 ± 0.2	10.1 ± 0.5	10.2 ± 0.6	5.6 ± 0.1
**11**	4.8 ± 0.4	9.2 ± 0.6	4.5 ± 0.6	4.3 ± 0.6	5.0 ± 0.1
**Auranofin**	3.4 ± 0.9	2.1 ± 1.0	2.5 ± 0.5	2.1 ± 0.7	<0.5

**Table 4:** Cytotoxicity (IC_50_ values in μM) of Au(I)-phosphane complexes.

Complex	A2780	A2780cis
**12**	0.051 ± 0.005	0.082 ± 0.031
**13**	0.023 ± 0.002	0.038 ± 0.013
**14**	0.133 ± 0.029	0.145 ± 0.038
**15**	0.106 ± 0.007	0.190 ± 0.076
**CDDP**	0.560 ± 0.140	5.352 ± 0.011

**Table 5:** IC_50_ (μM) values determined for gold(I) complexes **16**–**21**.

Complex	Hela 229	MCF-7	NCI-H460
**16**	1.79 ± 0.04	21.3 ± 0.7	10.4 ± 0.9
**17**	65 ± 1	76 ± 1	45 ± 1
**18**	69 ± 1	81 ± 1	N.D.
**19**	5.58 ± 0.17	29.2 ± 0.4	14.1 ± 0.3
**20**	8.54 ± 0.09	36.3 ± 0.2	22.3 ± 0.4
**21**	46 ± 0.11	72 ± 1	N.D.
Cisplatin	0.96 ± 0.02	13 ± 1	7.71 ± 0.67

N.D. = Not Determined.

**Table 6:** IC_50_ (µM) values of gold complexes **22** and **23**.

Complex	Incubation Time (h)	SH-SY5Y
**22**	6	6.2
	24	2.7
**23**	6	3.1
	24	1.6

**Table 7:** Cytotoxicity (IC_50_ µM) of complexes **24** and **25**.

Complex	Caco-2	MCF-7	Differentiated Caco-2	MDA-231
**24**	2.23 ± 0.21	0.46 ± 0.56	39.40 ± 23.39	0.78 ± 0.20 *
**25**	3.75 ± 0.41	3.53 ± 0.52	13.53 ± 0.02	0.27 ± 0.05 *

(*) *p* < 0.05 vs. free ligand.

**Table 8:** IC_50_ (µM) values of complexes **26** and **27**.

Complex	A17	MDA-MB-231	MDA-MB-468	HMLE/FoxQ1
**26**	11.38 ± 1.05	19.28 ± 1.06	13.62 ± 1.05	7.41 ± 1.06
**27**	11.95 ± 1.04	14.83 ± 1.05	11.25 ± 1.14	9.27 ± 1.06
**Cisplatin**	15.86 ± 1.17	50.49 ± 2	32.50 ± 1.12	34.12 ± 2

**Table 9:** IC_50_ (μM) values of complexes **28** and **29**.

Complex	HT29	HCT-116wt	HCT116-p53	DLD-1	518A2	MCF-7Topo
**28**	2.1 ± 0.1	6.8 ± 0.5	3.6 ± 0.1	3.8 ± 0.1	4.7 ± 0.2	4.1 ± 0.3
**29**	6.2 ± 0.1	6.7 ± 0.3	4.0 ± 0.1	3.6 ± 0.01	1.1 ± 0.1	7.0 ± 1.0

**Table 10:** IC_50_ (μM) values of complexes **30**–**36** against A549 cells.

Complex	30	31	32	33	34	35	36
Anticancer activity	13.32 ± 0.51	>100	11.91 ± 1.54	10.27 ± 0.76	10.61 ± 0.93	12.28 ± 2.66	9.11 ± 1.93

**Table 11:** IC_50_ values (µM) of complex **37**, erlotinib, and auranofin in cancer cells.

Complex	HT-29	MCF-7	MDA-MBA-231
**37**	3.90 ± 0.12	2.26 ± 0.17	1.64 ± 0.13
Erlotinib	>100	>100	68.11 ± 11.15
Auranofin	3.79 ± 0.18	2.00 ± 0.05	1.54 ± 0.12

**Table 12:** IC_50_ (µM) values of the complexes **41** and **42**.

Complex	Caco-2/TC7	MCF-7	HepG2	Dif Caco-2
**41**	1.52 ± 0.91	13.87 ± 0.78	3.38 ± 0.07	43.89 ± 0.02
**42**	2.33 ± 1.26	7.57 ± 0.08	5.88 ± 0.04	25.46 ± 0.66

**Table 13:** Cytotoxicity (IC_50_ µM) of complexes **43**–**45**.

Complex	PC-3	HEPG-2	MOLT-4	MCF-7
**43**	227.63 ± 0.06	245.78 ± 0.15	269.02 ± 0.10	283.48 ± 0.13
**44**	27.46 ± 0.01	26.01 ± 0.03	25.91 ± 0.03	22.58 ± 0.03
**45**	27.31 ± 0.01	27.94 ± 0.03	20.28 ± 0.03	18.63 ± 0.03
**Cisplatin**	39.99 ± 0.05	-	-	16.00 ± 0.06

**Table 14:** IC_50_ (µM) value of complexes **46**–**49** and cisplatin against cancer cell lines.

Complex	Hela	HepG-2	PC12	A549
**46**	1.74 ± 0.04	>100	4.75 ± 0.10	21.91 ± 0.27
**47**	1.93 ± 0.08	9.82 ± 0.07	2.04 ± 0.07	3.73 ± 0.43
**48**	7.25 ± 0.03	62.62 ± 0.08	3.06 ± 0.21	0.69 ± 0.19
**49**	9.47 ± 0.05	17.37 ± 0.05	5.61 ± 0.06	0.02 ± 0.01
**Cisplatin**	3.16 ± 0.36	9.80 ± 0.28	3.01 ± 2.91	1.12 ± 0.12

**Table 15:** IC_50_ (µM) values of **53** against the cancer and non-cancer cell lines.

Complex	A549	H1975	HEK-293
**53**	0.89 ± 0.08	0.23 ± 0.045	5.43 ± 0.28

**Table 16:** IC_50_ (µM) value of complexes **55**–**58** against cancer cell lines.

Complex	HCT-116	HT-116P	518A2	HeLa	KB-V1	HDFa
**55**	0.2 ± 0.02	0.05 ± 0.001	0.4 ± 0.1	0.3 ± 0.02	4.6 ± 0.2	1.4 ± 0.2
**56**	0.3 ± 0.03	0.2 ± 0.05	5.5 ± 0.4	3.6 ± 0.4	0.7 ± 0.2	3.2 ± 0.4
**57**	1.1 ± 0.3	0.6 ± 0.1	5.0 ± 0.3	3.6 ± 0.7	0.6 ± 0.2	5.8 ± 0.9
**58**	1.3 ± 0.6	0.4 ± 0.1	2.9 ± 0.5	1.8 ± 0.4	2.2 ± 0.2	5.9 ± 0.2

**Table 17:** Cytotoxicity (IC_50_ µM) of complexes **59** and **60**.

Complex	PC3	U87MG	HeLa	HT1080	SKOV-3	Hek-293T
**59**	22.5 ± 1.94	7.31 ± 0.72	13.8 ± 1.16	7.32 ± 0.85	18.3 ± 3.42	18.9 ± 2.82
**60**	1.96 ± 0.36	2.62 ± 0.24	8.7 ± 1.23	6.03 ± 0.51	10.3 ± 2.31	12.3 ± 0.81

**Table 18:** IC_50_ (µM) values of gold(I) complexes **61**–**64** towards cancerous cell lines.

Complex	Hela	MCF-7	SGC-7901
**61**	17.01 ± 3.29	24.15 ± 1.77	24.23 ± 7.44
**62**	19.27 ± 14.97	19.35 ± 11.67	18.52 ± 8.82
**63**	8.06 ± 0.63	20.35 ± 10.39	16.43 ± 1.86
**64**	17.75 ± 14.30	32.20 ± 31.11	24.71 ± 16.90

**Table 19:** IC_50_ (µM) value of complexes **65**–**67** against cancer cell lines.

Complex	A-549	HT-29	MDA-MB-231
**65**	3.0 ± 0.2	1.6 ± 0.1	0.8 ± 0.1
**66**	2.2 ± 0.7	1.0 ± 0.3	0.7 ± 0.2
**67**	2.3 ± 0.8	1.2 ± 0.6	1.0 ± 0.4

**Table 20:** Cytotoxicity (GI_50_ µM) * of complexes **68**, **69**, auranofin, and DHA.

Complex	A549	U-2 OS	MCF-7	T24	LAMA	HL-60	HepG2
**68**	0.115	0.122	0.089	0.175	0.079	0.017	2.16
**69**	1.16	2.51	0.380	0.191	0.662	0.500	5.23
**Auranofin**	4.41	0.474	1.39	1.10	0.809	0.951	3.62
**DHA**	11.1	4.10	9.67	4.99	5.60	3.25	12.0

* The GI50 values represent the concentration of compounds causing 50% inhibition of cell growth. Mean of at least three independent experiments.

**Table 21:** IC_50_ (µM) values of complexes **70**–**72**.

Complex	HCT-116^wt^	MCF-7^topo^
**70**	0.64 ± 0.01	1.1 ± 0.2
**71**	0.41 ± 0.01	0.70 ± 0.06
**72**	0.29 ± 0.01	0.80 ± 0.04

**Table 22:** IC_50_ (µM) values of the complex **73**.

Complex	HeLa	MCF-7	V79
**73**	7.26 ± 4.3	7.92 ± 1.0	63.7 ± 8.6

**Table 23:** IC_50_ (µM) values of complexes **74** and **75**.

Complex	MCF-7	MDA-MB-231	HT-29
**74**	3.7 ± 0.7	5.2 ± 0.8	6.3 ± 1.8
**75**	4.0 ± 1.4	11.0 ± 1.1	10.6 ± 2.8
**Cisplatin**	2.6 ± 0.5	2.7 ± 0.9	6.9 ± 1.8
**Auranofin**	0.6 ± 0.2	2.1 ± 0.7	3.3 ± 0.0

**Table 24:** Cytotoxic activity (IC_50_ µM) values of gold(I) NHC complexes **76** and **77**.

Complex	A2780cis	A2780	HepG2	HepAD38	MDCK
**76**	0.11 ± 0.02	0.67 ± 0.3	0.46 ± 0.12	0.39 ± 0.13	3.0 ± 0.5
**77**	0.36 ± 0.1	0.17 ± 0.04	0.97 ± 0.14	1.3 ± 0.2	2.1 ± 0.6
**Cisplatin**	34 ± 2.4	3.2 ± 0.61	4.31 ± 1.10	12 ± 1.3	27 ± 3.8

**Table 25:** Cytotoxicity (IC_50_ µM) of complex **78**, auranofin, and cDDp.

Complex	TrxR	SW480	A549	HepG2
**78**	6 ± 1	10 ± 1	20 ± 2	9 ± 1
**Auranofin**	-	0.7 ± 0.1	-	1.2 ± 0.1
**cDDP**	-	47 ± 1	38 ± 2	29 ± 1

**Table 26:** Cytotoxicity (IC_50_ µM) of complexes **79** and **80**.

Complex	MCF-7	MDA-MB-231	HT-29
**79**	4.0 ± 1.4	11.0 ± 1.1	10.6 ± 2.8
**80**	3.7 ± 0.7	5.2 ± 0.8	6.3 ± 1.8

**Table 27:** IC_50_ (µM) value of complexes **81**–**83** against cancer cell lines.

Complex	THP1
**81**	15.84 ± 2.86
**82**	16.24 ± 3.27
**83**	20.93 ± 1.68
**Miltefosine**	35.02 ± 2.98

**Table 28:** IC_50_ (µM) values for NHC complexes **84** and **85**.

Complex	HEP3B	SHSY5Y	HF
**84**	9.32 ± 0.95	55.16 ± 5.28	108.5 ± 9.34
**85**	10.51 ± 1.01	102.5 ± 11.8	215.7 ± 16.6

**Table 29:** IC_50_ (µM) values for NHC complexes **86**.

Complex	86	86 + Pd4′	86 + Pd5
**A549**	63.3 ± 1.1	13.1 ± 2.8	13.1 ± 4.0
**HepG2**	70.6 ± 0.7	12.0 ± 3.6	18.5 ± 1.2
**MCF-7**	54.3 ± 0.2	11.8 ± 1.9	13.8 ± 0.3
**U87**	52.4 ± 0.2	8.5 ± 0.2	6.4 ± 1.8

**Table 30:** IC_50_ (µM) values of metal complexes **90**–**92** and cisplatin.

Complex	MCF-7	MDA-MB 231	HeLa	ISHIKAWA	MCF-10A	Hek293
**90**	18.2 ± 0.5	31.7 ± 0.8	44.5 ± 0.9	54.9 ± 1.0	>200	>200
**91**	9.38 ± 0.2	53.7 ± 0.7	98.6 ± 1.2	12.6 ± 0.3	158.8 ± 1.0	>200
**92**	5.18 ± 0.4	2.1 ± 0.7	31.9 ± 0.5	29.9 ± 0.9	>200	65.0 ± 0.7
**Cisplatin**	35.8 ± 1.3	28.7 ± 1.0	15.7 ± 1.1	15.1 ± 0.8	81.3 ± 1.2	16.8 ± 0.4

**Table 31:** IC_50_ (μM) values of complex **94**.

Complex	A549-24 h	A549-72 h	HeLa-24 h	HeLa-72 h
**94**	>25	17.52 ± 1.40	18.8 ± 1.15	2.35 ± 0.06

**Table 32:** In vitro cytotoxic activity (IC_50_ μM) of gold complexes **96** and **97**.

Complex	A549	SKOV3	MCF-7	MCF-10A
**96**	19.46 ± 1.15	11.76 ± 1.49	13.27 ± 3.37	47.16 ± 1.28
**97**	32.75 ± 1.47	22.52 ± 2.23	19.14 ± 1.28	45.08 ± 2.61

**Table 33:** IC_50_ (µM) value of complexes **98**–**100**.

Complex	HeLa	A549	HT1080	Caov-3
**98**	0.3 ± 0.2	0.07 ± 0.06	0.14 ± 0.04	0.3 ± 0.2
**99**	0.6 ± 0.2	4.5 ± 0.7	4.4 ± 1.3	3.9 ± 0.8
**100**	2.7 ± 0.1	6.6 ± 2.5	3.1 ± 1.8	1.9 ± 0.4

**Table 34:** IC_50_ values of gold(I) complexes **101**, **102**, cisplatin, and auranofin.

Complex	MDA-MB-231	HT-29	MCF-10A	MCF-7
**101**	3.18 ± 0.05	2.32 ± 0.10	67.65 ± 0.52	3.42 ± 0.15
**102**	1.97 ± 0.10	0.98 ± 0.05	89.25 ± 0.42	1.25 ± 0.05
**Cisplatin**	7.41 ± 0.12	4.17 ± 0.25	35.42 ± 0.32	2.17 ± 0.40
**Auranofin**	5.14 ± 0.13	2.94 ± 0.12	47.25 ± 0.60	1.82 ± 0.12

**Table 35:** The cytotoxic activity of cisplatin, copper-based complexes, and gold-based complexes was evaluated in IGROV-1 cells.

Complex	IGROV-1
**103**	27.5 ± 3.5
**104**	8.3 ± 3.2
**Cisplatin**	6.8 ± 0.6

**Table 36:** Summary of clinical studies evaluating gold-derived complexes in cancer.

S. No.	NCT Number	Study Focus	Disease/Cancer Type	Study Design	Numbers of Patient Enrolled	Start Year	Study Outcomes	References
**1.**	NCT02770378	Multi-drug regimen including standard chemotherapy	Glioblastoma	Interventional	10	2016	Evaluated the feasibility and therapeutic response in glioblastoma	[93]
**2.**	NCT01737502	Combination therapy using Auranofin and sirolimus	Small Cell Lung Cancer (SCLC)	Phase I/II, interventional	29	2014	Evaluated safety, tolerability, and preliminary efficacy of combination therapy	[94]
**3.**	NCT01747798	Auranofin as monotherapy	Recurrent ovarian/fallopian tube cancer	Phase II, interventional	10	2012	Assessed antitumor activity and safety profile	[95]
**4.**	NCT01419691	Auranofin in hematological malignancy	Chronic Lymphocytic Leukemia (CLL)	Phase I/II, interventional	15	2011	Determined safety, dosing, and preliminary efficacy	[96]
**5.**	NCT00575393	Gold sodium thiomalate therapy	Lung cancer	Phase I, interventional	17	2007	Assessed safety and early clinical response	[97]

## Data Availability

No new data were created or analyzed in this study. Data sharing is not applicable to this article.

## Abbreviations

Au	Gold
BTC	Breast tumor cells
CAAC	Cyclic(alkyl)(amino)carbene
CDDP	Cis-diamminedichloroplatinum (II) or Cisplatin
CLL	Chronic lymphocytic leukemia
HDFa	Human adult dermal fibroblast cells
HEK	Human embryonic kidney cell
HF	Human fibroblasts
NHC	*N*-heterocyclic carbene
NSCLC	Non-small cell lung cancer
SCLC	Small cell lung cancer
THP	Tris(hydroxymethyl) phosphane
TrxR	Thioredoxin reductase
